# Identification of native protein structures captured by principal interactions

**DOI:** 10.1186/s12859-019-3186-6

**Published:** 2019-11-21

**Authors:** Mehdi Mirzaie

**Affiliations:** Department of Applied Mathematics, Faculty of Mathematical Sciences, Tarbiat Modares University, Jalal Ale Ahmad Highway, P.O.Box: 14115-134, Tehran, Iran

**Keywords:** Knowledge-based potential, Principal interaction, Native structure, Decoy set

## Abstract

**Background:**

Evaluation of protein structure is based on trustworthy potential function. The total potential of a protein structure is approximated as the summation of all pair-wise interaction potentials. Knowledge-based potentials (KBP) are one type of potential functions derived by known experimentally determined protein structures. Although several KBP functions with different methods have been introduced, the key interactions that capture the total potential have not studied yet.

**Results:**

In this study, we seek the interaction types that preserve as much of the total potential as possible. We employ a procedure based on the principal component analysis (PCA) to extract the significant and key interactions in native protein structures. We call these interactions as principal interactions and show that the results of the model that considers only these interactions are very close to the full interaction model that considers all interactions in protein fold recognition. In fact, the principal interactions maintain the discriminative power of the full interaction model. This method was evaluated on 3 KBPs with different contact definitions and thresholds of distance and revealed that their corresponding principal interactions are very similar and have a lot in common. Additionally, the principal interactions consisted of 20 % of the full interactions on average, and they are between residues, which are considered important in protein folding.

**Conclusions:**

This work shows that all interaction types are not equally important in discrimination of native structure. The results of the reduced model based on principal interactions that were very close to the full interaction model suggest that a new strategy is needed to capture the role of remaining interactions (non-principal interactions) to improve the power of knowledge-based potential functions.

## Background

The protein structure is a result of non-covalent interactions between its residues in three-dimensional space. One of the key challenges in structural bioinformatics is to promote an understanding of the structure of the complex network of non-covalent interactions in proteins that significantly contribute to the three-dimensional structure [1]. Because of its complexity, the structure and the amino acid sequence of proteins need to simplify to make analysis tractable [2]. Protein folds into a single three-dimensional conformation among an astronomical number of possible conformations in order of microseconds. According to Levinthal paradox [3], a protein could not exhaustively search all possible conformational states to achieve the native structure. Despite the advances in experimental and computational methods in protein structure analysis, the problem of folding protein has not solved yet.

Amino acids are the building blocks and the basic components of proteins. Anfinsen [4] believed that information of amino acid sequence is sufficient to determine the native fold of a protein. Several studies with different computational and experimental approaches suggested reduced alphabet of amino acids in protein design, protein fold recognition [5–9]. Reduced models of protein structure could decrease the complexity of the protein folding problem and be useful in protein design [5]. Therefore the determination of essential amino acids and their interactions in native protein structures is an important issue that needs to be addressed.

According to Anfinsen thermodynamic hypothesis, the native structure of a protein corresponds to minimum free energy [4]. For a given amino acid sequence, the protein structure prediction problem is to find a three-dimensional structure such that the total free energy is minimized. Therefore, the assessment of protein structure needs a trustworthy potential function. Knowledge-based potentials (KBPs) [6, 7] as an approximation of the free energy are extracted from the analysis of experimentally determined protein structures. They are mainly based on the distribution of a feature relative to a reference state [8]. There are various KBP including contact potentials [6, 9], orientation-dependent potentials [10–15], distance-dependent potentials [16–20], multi-body potentials [21]. Impressive progresses in protein design [22], simulation of protein folding [23], ligand binding [24, 25], aggregation [26], protein structure prediction [27], and fold recognition [28] have been achieved using knowledge-based potential functions.

In the previous work [29], we reported the importance of hydrophobic non-local interactions in scoring function (based on Delaunay tessellation) in discrimination of native structures. Here, in order to reduce protein structure, we employ a PCA-based way to formulate and extract principal interactions in protein 3D structure. The PCA (Principal Component Analysis) technique was first introduced for only a few variables in 1901 by Karl Pearson [30] and then it was extended by Hotelling in 1933 for a large number of variables [31]. Today, PCA is a technique which has been widely used to select the most important variables from a multivariate data and retrieve dominant pattern from noisy data. In fact, PCA maps a complex system from multidimensional space to a reduced space spanned by a few principal components to clarify the principal features underlying the observed data [32]. The three-dimensional structure of a protein could be considered as such multidimensional data [33]. Studies such as protein dynamics prediction from experimental data [34], discovery of binding site [35], prediction of protein intermediate states [36], protein folding dynamics [37], analysis of molecular dynamics trajectories [38], and principal components analysis of protein structure ensembles [33, 39] are a few examples among many applications of PCA in protein structure analysis. If the native structure of a protein lies in the minimum potential and the total potential approximated by a summation of pair-wise interaction type energies, we assert that the goal of structure reduction should be to preserve as much of total energy as possible. PCA is a natural tool for extracting these most important variables (pair-wise interaction types) in total potential. The presented method extracts significant (principal) interactions by considering only native conformations without using decoys. We show that the principal interactions perform well in the identification of native structure, close to the same discriminatory power as the full interaction model. Our model derives the observation that principal interactions extracted in the three-dimensional structure of native structures will tend to dictate a minimum energy protein conformation among decoy structures, suggesting that these gross features are dominant in dictating the structure.

In this study, at first, the energy between all pair of residues using distance dependent knowledge-based potential function for each native structure was calculated. Since there is 210 possible amino acid-amino acid interaction type between 20 amino acids, therefore for each protein structure we could have a vector with 210 elements, containing energy between amino acid-amino acid interaction types, where for example i’th element is the summation of energy between all ALA-GLY residues in the structure. We obtained a (*n* sample) × (210 variables) matrix where *n* is the number of native protein structures in the train dataset. Finally, the principal variables (interactions) were extracted using a PCA-based approach. To our astonishment, these interactions that consisted of 20% of the full interaction on average, were found between residues that are known as critical, important, and effective in protein folding. The model that considers only the principal interactions was assessed by six measures including the number of correctly identified natives, the Z-score of the native energy, the RMSD of the minimum energy, the Pearson correlation coefficient between energy and Cα RMSD from the native structure, 20% fraction enrichment and the Z-score of the best decoy structure using two decoy sets. The results are very similar and close to the full interaction model. Therefore, we are able to demonstrate that the principal interactions maintain the discriminative power of the full interaction model. Additionally, the similar results were found when the principal interaction model was evaluated on DOPE and DFIRE potential functions.

## Results

### Principal interactions of protein native structures

The general definition of distance-dependent knowledge-based potential between pairs of atoms *i* and *j* at distance *d* is:

1 $$ \Delta  {E}^{ij}(d)=- RTln\frac{f_{obs}^{ij}(d)}{f_r^{ij}(d)}, $$

where ${f}_{obs}^{ij}(d)$ and ${f}_r^{ij}(d)$ represent the relative frequency of atomic pairs *i* and *j* at distance *d* in a database of native structures and the reference state, respectively [7]. The total potential of a given protein structure, S, denoted by *∆E*(*S*) can be approximated as follows [7]:

2 $$ \Delta  E(S)=\frac{1}{2}{\sum}_{i,j}\Delta  {E}^{ij}(d). $$

The potential between pairs of residues *A* and *B* can be calculated as a summation of pairwise potentials between atoms of residue *A* and *B*:

3 $$ \Delta  E\left(A,B\right)=\sum \limits_{i\in A,j\in B}\Delta  {E}^{ij}(d), $$

where i and j are atoms of residues *A* and *B*. Therefore, the total potential of a protein structure, S, can be approximated as follows:

4 $$ \Delta  E(S)=\frac{1}{2}{\sum}_{A,B\in T}\Delta  E\left(A,B\right) $$

where T is set of all 20 amino acid types. Since there are 210 possible different amino acid-amino acid interaction types between 20 amino acids, the total number of distinct *∆E*(*A*, *B*) in the above summation is equal to 210.

A non-redundant structural dataset of 6944 protein chains was culled by PISCES [40] from Protein Data Bank with pairwise sequence identity < 50%, resolution < 1.6 Å, R-factor < 0.25, protein length > 40 and < 1000 residues. We also excluded the proteins with more than 50% sequence identity with targets in test sets (proteins in the 3DRobot [41] and CASP10–13 [19]). The final list contains 6384 protein chains. The final list of PDB and chain IDs are provided in Additional file 1: Table S1.

Let S_i_ be i’th protein native structure in the data set and the 210-dimensional vector P_i_, containing 210 amino acid-amino acid energies; *∆E*(*A*, *B*) as explained above. Obviously, the sum of P_i_ is equal to *∆E*(*S*_*i*_); the total energy of the protein structure S_i_. The total energy matrix (denoted by TE) is a 6384 sample-by-210 variables matrix whose rows are P_i_ calculated from 6384 native protein structures.

**Fig. 1:**
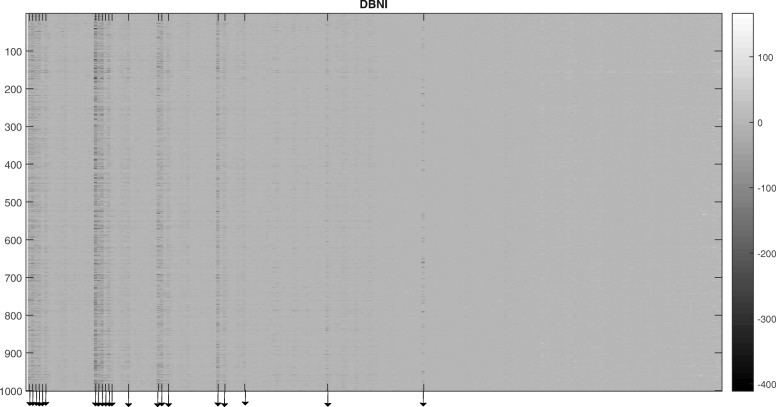
A color image representation of the CTE matrix for DBNI. The x-axis represents 210 variables and the y-axis represents1000 randomly selected protein structures from train data set. The extracted principal interaction positions on the x-axis are shown by arrows. (From left to right: F-F, F-L, F-I, F-V, F-W, F-Y, L-L, L-I, L-V, L-W,L-Y, L-M, L-A, I-I, I-V, I-Y, V-V, V-Y, V-A, Y-Y, and C-C, respectively)

At first, each of the samples in TE matrix has been centered to have mean zero, the new matrix is called CTE (centered total energy). Figure 1 shows the image representation of CTE matrix for DBNI scoring function [42]. In DBNI, the neighbors of atoms (interactions) are determined by Delaunay tessellation and only non-local interactions (those between atoms farther than five residues in the sequence) by a distance less than 6 Å are considered. In fact, the figure displays the data matrix CTE as an image that uses the range of colors as indicated in the colorbar. Each element of CTE specifies the color for one pixel of the image. The smallest value in CTE is mapped into the black color. As shown in Fig. 1, the values of some variables or interactions shown as black points are very low and different from the others.

The extracted principal interactions (variables) as described in Methods section are as follows: LEU-LEU, CYS-CYS, PHE-LEU, VAL-VAL, ILE-LEU, PHE-PHE, ILE-ILE, LEU-VAL, ILE-VAL, LEU-TYR, PHE-ILE, PHE-VAL, TYR-TYR, LEU-TRP, ILE-TYR, LEU-ALA, PHE-TYR, VAL-TYR, VAL-ALA, TRP-PHE, and MET-LEU. These interactions are shown by arrows in Fig. 1. Most of these interactions are between amino-acids that are critical and important in protein structure and protein folding. Val, Ala, Leu, Ile, Phe, Tyr, and Trp are hydrophobic amino acids and are important in determining the three-dimensional structure of proteins. Tyrosine is an aromatic, partially hydrophobic amino acid that is inclined to be buried in protein hydrophobic cores. The aromatic side-chain can also mean that tyrosine is involved in stacking interactions with other aromatic side-chains such as phenylalanine and tyrosine. Cysteine has sulfur-containing side group with the potential to be more reactive. As a group with low polarity, cysteine is known for its ability to connect with another cysteine via the sulfur atoms to form a covalent disulfide bridge, which is important in the formation and maintenance of the tertiary (folded) structure in many proteins. In order to assess the principal interactions in the identification of the native structure, the results of the *full interaction model* were compared with the *principal interaction model*. In the *full interaction model,* all amino acids and their interactions, as determined in their corresponding KBP definition, are considered. In comparison, in the *principal interaction* model*,* only the principal interaction types of the *full interaction model* are considered.

### Validation of the *principal interaction* model on decoy sets

The *principal interaction model* was assessed in discrimination of native structure, using the decoy sets *3DRobot* [41] *and CASP10–13* [19]. The *3DRobot* set includes decoy structures for 200 non-homologous proteins randomly selected from the PDB library. This protein set contains 48 α-, 40 β-, and 112 α/β-single-domain proteins with lengths ranging from 80 to 250 residues. Each protein contains 300 structural decoys with RMSD ranging from 0 to 12 Å. The *CASP10–13* decoy set includes a total of 13,474 structures for 175 proteins, which were collected and trimmed from CASP10-CASP13 experiments by Yu et.al [19]. In this data set, all prediction sets contain experimental structures that are sequentially consecutive and all non-first prediction models (the second to fifth models of predictors) have been removed. In addition, the selected prediction models are also consecutive and identical in sequence to the corresponding experimental structure. This data set has many decoys with RMSDs evenly distributed from very similar to very different to the native structure. The model was assessed using six measures including, the number of correctly identified native structures (Top1), the Z-score of the native structure energy, the RMSD of the minimum energy, the Pearson correlation coefficient (denoted by PC) between RMSD from the native structure and the total energies, 20% fraction enrichment, and the Z-score with respect to the best decoy as explained in the Methods section.

In order to compare the difference between the *principal interaction model* and the *full interaction model* in size, the percentage of principal interactions denoted by PI for all decoy structures in the data sets was also calculated:

$$ \mathrm{PI}=\left(\#\mathrm{principal}\ \mathrm{interactions}/\#\mathrm{full}\ \mathrm{interactions}\right) $$

A comparison of two models using DBNI potential function is summarized in Table 1. The values in parenthesis depict the performance of the potential function in the *full interaction model*. The PI values represents the average of the percentage of interactions between atoms used for the calculation of energy in the *principal interaction model*. On average, 25% of interactions are used in the *principal interaction model*.

**Table 1:** Assessment of DBNI scoring function on the *principal interaction model* and the *full interaction model* on Decoy Sets

Decoy Sets	CASP10–13	3DRobot	Total/Average
Top1	109 (107)	177 (179)	286 (286)
Z-score	1.46 (1.32)	4.07 (4.36)	2.76 (2.84)
RMSD	4.07 (4.26)	0.19 (0.19)	2.13 (2.22)
PC	0.13 (0.12)	0.50 (0.58)	0.31 (0.35)
F.E.	1.30 (1.20)	2.95 (3.14)	2.12 (2.17)
Z-score_b_	0.25 (0.29)	1.60 (1.87)	0.92 (1.08)
PI	0.26 ± 0.05	0.25 ± 0.05	0.25 ± 0.05
#Target	175	200	375

The Top1 is the number of first-ranked native structures within the decoy sets. The Z-score is the average of Z-score of the native energy, the RMSD represents the average of the RMSD of the minimum score, and the PC is the average of Pearson correlation coefficient between the energy and RMSD from the native structure. The PI represents the average of the percentage of interactions between atoms used for the calculation of energy in the *principal interaction model*. The F.E. indicates the 20% fraction enrichment, and the Z-score_b_ calculates the Z-score with respect to the best decoy. The last row is the number of targets in decoy sets. The values in the columns denote the performance of potential functions in the *principal interaction model* and the values in parenthesis depict the performance of the potential function in the *full interaction model*

The *principal interaction model* and the *full interaction model* correctly identified 286 native structures with a success rate of 76%. The results of the *principal interaction model* in all six criteria are very similar to the *full interaction model*.

The F.E. and Z-score_b_ evaluate the performance of the model in decoy discrimination between good and bad structural decoys. The possible values of F.E. ranges from 0 to 5 [19]. The higher value of F.E. the better decoy discrimination. For 3DRobot, the results of F.E. and Z-score_b_ are better than CASP10–13. Because most of the targets in 3DRobot have decoy structures with low RMSD, however in CASP10–13 the minimum RMSD is 5 Å for 44% of the targets. For *3DRobot* decoy set, all criteria justify the model. The averages of CC’s in the *principal interaction model* and the *full interaction model* are 0.50 and 0.58, respectively with the average of Z-scores higher than 3. For both decoy sets, the results of the *principal interaction model* are very similar and close to the *full interaction model* using 25% of the full interactions on average and adding further down the list of interactions could not significantly increase the performance of the potential function.

## Discussion

### Principal interaction model on DFIRE and DOPE

**Fig. 2:**
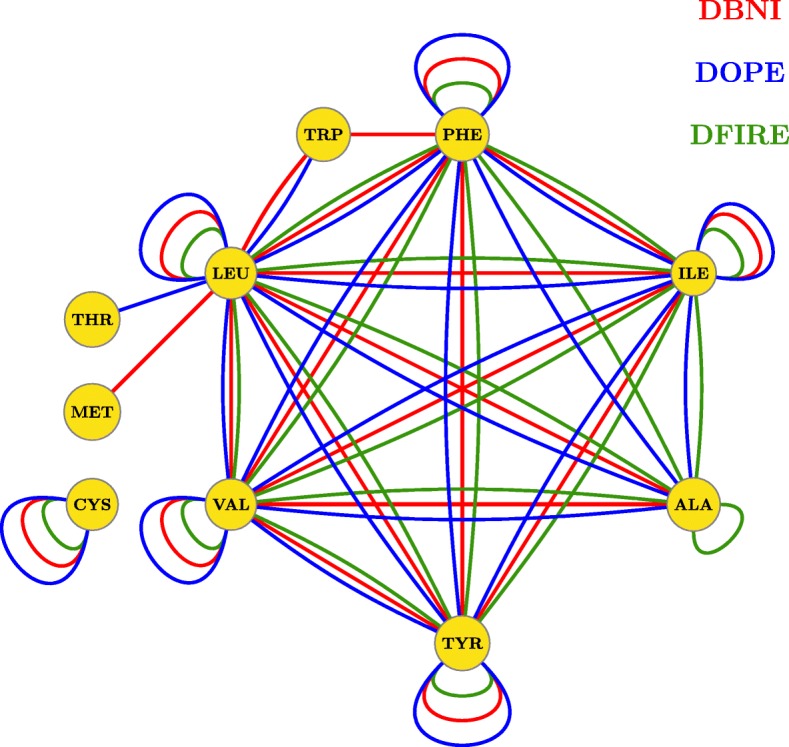
The principal interactions in DBNI, DOPE, and DFIRE are represented by red, blue and green edges, respectively

**Fig. 3:**
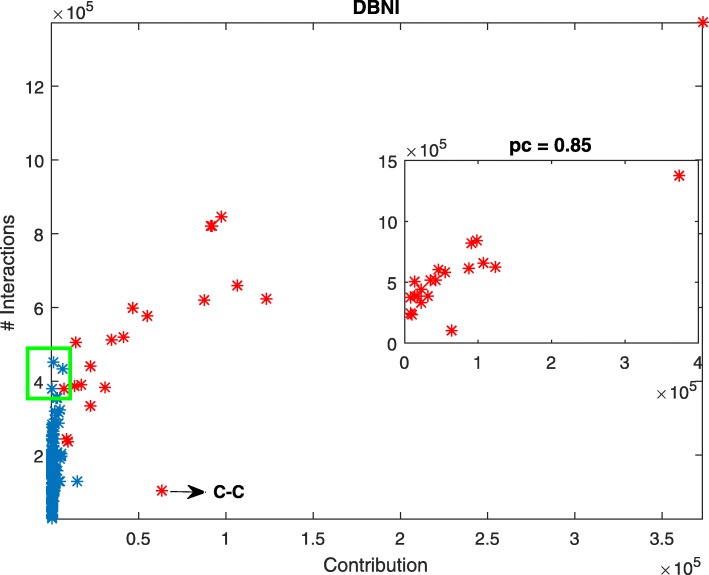
The number of pairwise interactions versus their contribution values for DBNI. The right-hand side inset plot presents the relation between the principal interactions and their associated contribution value

The *principal interaction model* was also tested on DFIRE [43], and DOPE [17] potential functions. In DBNI, atomic interactions are determined by Delaunay tessellation and only non-local interactions (those between atoms farther than five residues in the sequence) by a distance less than 6 Å are considered. DOPE and DFIRE are derived from a non-interacting ideal gas reference state, except that in DOPE the size effect of proteins is taken into account. In DFIRE and DOPE, two atoms by a distance less than 15 Å are in contact. The image representation of the CTE matrix for DFIRE and DOPE are shown in the Additional file 1: Figure S1 and S2. The extracted principal interactions (variables) for these potential functions are shown in Fig. 2 by red, black, and blue edges, respectively, and are listed in Additional file 1: Table S2. Although the aforementioned potential functions have differences in the definition of contact, the threshold of distance, and ignorance of local interactions, most of their principal interactions, as shown in Fig. 2, are the same. Note that these interactions are between residues considered as important in protein folding. For each interaction type, the number and the contribution value were calculated. The number of interactions was extracted from train data set and the contribution value was calculated by applying the procedure described in the Methods section on CTE matrix whose columns are pairwise potentials calculated from 6384 native structures in the train set. In summary, at first the covariance matrix of the CTE matrix was calculated using Eq. 5 and then it diagonalized according to Eq. 6. As described in the Methods section, the set of *m* eigenvectors of the covariance matrix (V_1_,V_2_, …,V_m_) with 80% of total variations was selected (Eqs. 7 and 8). The contribution of the pairwise interaction *k*, in a given principal direction V_j_ denoted by C_kj_ was calculated in Eq. 9. Let C_k1_, C_k2_, …, C_km_ is the contributions of the pairwise interaction *k* on V_1_,V_2_, …, V_m_, respectively. The *total contribution* of the pairwise interaction *k* was calculated using Eq. 10. Figure 3 shows the relation between the number of interaction and the contribution value for DBNI potential function and the right-hand side inset plot corresponds to only the principal interactions. As shown in the figure, the principal interactions indicated by red points mainly have a large number of interactions, however the number of interactions for CYS-CYS (inferred as principal interaction and indicated by arrow in the figure) is relatively low and on the other hand, the non-principal interactions such as ARG-LEU, GLU-ARG, and ASP-ARG (data points in the green rectangle, the bellow left-hand side) have a large number of interactions. The similar results obtained for DOPE and DFIRE and presented in Additional file 1: Figure S6 and S7.

The results for these well-designed potential functions, DFIRE, and DOPE are summarized in Table 2. The results of the *principal interaction model* for all these potential functions are also very close to the *full interaction model* and justify the principal interaction model well.

**Table 2:** Assessment of DFIRE and DOPE scoring functions on the *principal interaction model* and the *full interaction model* on Decoy Sets

Decoy Set	DFIRE			DOPE		
	CASP10–13	3DRobot	Total/Average	CASP10–13	3DRobot	Total/Average
Top1	54 (56)	13 (12)	67 (68)	75 (74)	63 (71)	138 (145)
Z-score	1.18 (1.18)	1.33 (1.20)	1.92 (1.19)	1.39 (1.37)	1.64 (1.90)	1.51 (1.63)
RMSD	6.95 (6.01)	2.02 (1.44)	4.48 (3.72)	5.55 (5.31)	1.28 (0.82)	3.41 (3.06)
PC	0.56 (0.51)	0.80 (0.81)	0.68 (0.66)	0.54 (0.44)	0.81 (0.82)	0.67 (0.63)
F.E.	1.59 (1.56)	3.73 (3.74)	2.66 (2.65)	1.63 (1.58)	3.86 (3.87)	2.74 (2.72)
Z-score_b_	0.54 (0.47)	1.45 (1.65)	1.00 (1.06)	0.53 (0.46)	1.54 (1.77)	1.03 (1.11)
PI	0.18 ± 0.05	0.19 ± 0.05	0.18 ± 0.05	0.19 ± 0.05	0.21 ± 0.05	0.20 ± 0.05
#Target	175	200	375	175	200	375

**Fig. 4:**
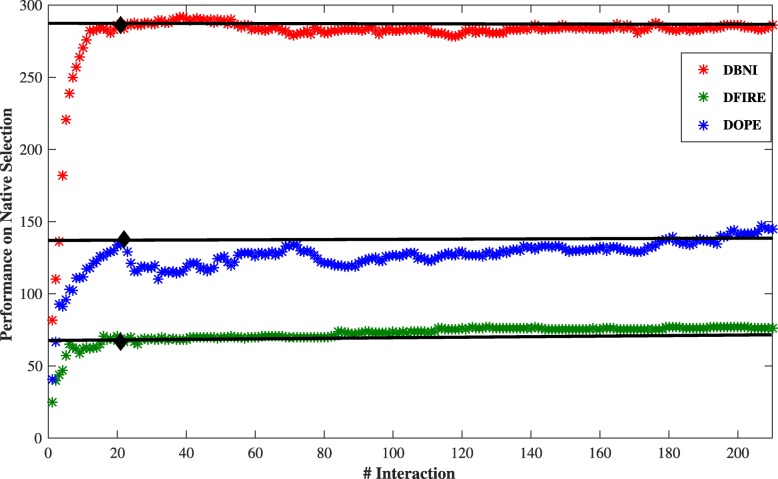
The success rate versus the index of sorted interactions according to their contribution

The success rate of three KBP functions in terms of the number of first-ranked native structures is shown in Fig. 4. The X-axis represents the index of sorted interactions based on their contribution values; the details are shown in Additional file 1: Table S2. The horizontal dashed line indicates the success rates obtained by the *principal interaction model*, which intersect the diagrams in bold points. The progress of the overall performances represents a sharp initial increase up to the bold points, followed by a straightening out or a slim decrease and increase. Therefore, the performance of these scoring functions is captured and achieved by the principal interactions.

## Conclusions

In this study, we showed that amino acids and their interactions are not equally important in protein structure. The method is relatively straightforward and easy to apply to other potential functions. The results of the reduced model based on principal interactions inferred using three knowledge-based potential functions reveal that non-principal interactions do not increase significantly the performance of the knowledge-based potential functions. Hence, a new strategy is needed to capture the role of polar and charged amino acids for improving knowledge-based potential functions. Additionally, the simplicity of the protein structure could help to disclose the hidden secret of protein folding. By reducing the 20 amino acids to the simplified alphabet, the degree of freedom of protein structure could be greatly reduced, and the main mechanism and the underlying rules could be decoded. An appropriate simplification should regenerate the structural features of proteins. It is generally assumed that native protein structure takes its conformation at minimum energy. The pairwise potential is a widely used measure to capture the strength of pairwise interactions. In the context of energy landscape theory, the energy of the simplified native structure should be also at the minimum on the landscape of the simplified version. The application of the principal interaction model in de novo design and threading approaches would be a future direction.

## Methods

### Principal interactions extraction

PCA is generally used to select a subset of variables from a large set while retaining most of the information. This is achieved by transforming the original variables to a new set of variables, the principal components (PCs), which are ordered so that the first few retain most of the variation present in all of the original variables and have the highest correlations with the principal components [44]. Let the data matrix to be analyzed by PCA comprises *n* samples described by *p* variables and represented by the *n* × *p* matrix *E,* whose j-th column is the vector x_j_ of samples on the j-th variable. In the mean centered data, which we write as *U,* the column means of *E* have been subtracted from their corresponding columns and so the column means of *U* are equal to zero. The covariance matrix of the mean centered data, *U*, could easily be calculated as follows [44]:

5 $$ \Sigma =\frac{U^TU}{n-1}, $$

It is a symmetric matrix and so it can be diagonalized:

6 $$ \Sigma = VL{V}^T, $$

where V is a matrix of eigenvectors (each column is an eigenvector) and L is a diagonal matrix with eigenvalues *λ*_*i*_ in the decreasing order on the diagonal. Each eigenvalue is proportional to the portion of the variance that is associated with each eigenvector. The eigenvectors are called principal axes or principal directions of the data. Let the principal directions of the covariance matrix Σ be V_1_, V_2_, …, V_p_ (these vectors are the columns of V) and λ_1_, λ_2_, …, λ_p_ be the eigenvalues of the covariance matrix. Here, we choose the set of *m* (*m ≤ p*) eigenvectors of Σ which have the *m* largest eigenvalues with 80% of total variations.

In order to assess the effect of each *∆E*(*A*, *B*) on the total potential of the mean force, *∆E*(*S*), we used the following procedure on the 500 × 210 matrix CTE:

1. Let *V*_*1*_*, V*_*2*_*, …, V*_*210*_ and λ_1_, λ_2_, …, λ_210_ be the eigenvectors (principal directions) and the eigenvalues (variances) of the covariance matrix of CTE. The covariance matrix is calculated using formula (3). For a given *V*_*j*_, the proportion of total variance that it accounts for is:

7 $$ {t}_j=\frac{\lambda_j}{\sum_{i=1}^{210}{\lambda}_i}\times 100, $$

The first *m* Vs (*V*_1_, *V*_2_, …, *V*_*m*_) were chosen such that the sum of the proportion of total variance be at least 80%. That means:

8 $$ \sum \limits_{j=1}^m{t}_j\ge 80\%, $$

Variables (interactions) that are correlated with *V*_1_, *V*_2_, …, *V*_*m*_ possess high contribution and we consider them as the most important variables (interactions). The *contributions* of the variable *k* in accounting for the variability in a given principal direction, *V*_*j*_, are calculated (in percentage) as:

9 $$ {C}_{kj}=\frac{V_j{(k)}^2}{\sum_{k=1}^{210}{V}_j{(k)}^2}\%, $$

where *V*_*j*_(*k*) is k-th element of *V*_*j*_

2) Let *C*_*k*1_, *C*_*k*2_, …, *C*_*km*_ be the contributions of variable *k* on *V*_1_, *V*_2_, …, *V*_*m*_, respectively. The *total contribution* of variable *k* on explaining the variations retained by *V*_1_, *V*_2_, …, *V*_*m*_ is calculated as:

10 $$ {\sum}_{i=1}^m\left({C}_{ik}\times {\lambda}_i\right). $$

The 210 variables are sorted by the values of the variable’s total contribution. The total contribution of a variable is compared to the average contribution of that variable to a uniform distribution of contributions as follows:

3) Assuming uniform contributions for the considered 210 variables, the contribution of a variable on a given direction would be $\frac{1}{210}\%$. In this case, the average contribution of a variable for *V*_1_, *V*_2_, …, *V*_*m*_ is: $\tau =\sum \limits_{i=1}^m\left(\frac{1}{2.1}\times {\lambda}_i\right)$
4) For the given *V*_1_, *V*_2_, …, *V*_*m*_, a variable with a contribution larger than this cut-off, *τ*, could be considered important and significant in contributing to the components. We call these interactions (variables) as the *principal interactions* (variables).

### Performance criteria

The performance criteria for model assessment and their definitions are as follows:

1. Top1: Native structure is correctly identified if its structure has the lowest value of energy. The number of correctly identified native structures in a decoy set is denoted by Top1.
2. Z-score of the native structure energy in a decoy set is as follows:
  $$ Z- score=\frac{\left\langle {score}_{decoys}\right\rangle -{score}_{native}}{\sigma_{decoys}} $$

where *score*_*native*_scorenative is the score (energy) calculated for a native structure, <*score*_*decoys*_ > and *σ*_*decoys*_ are the average and standard deviation of score distributions of decoys proteins, respectively.

3) RMSD of the minimum energy: The root mean square deviation of the Cα-Cα pairs between the native structure and the structure with the minimum energy.
4) PC: The Pearson correlation coefficient between Cα RMSD from the native structure and the total energies.
5) Z-score_b_: The energy gap between the *best* decoy structure (the model with lowest RMSD from the native structure) and the remaining structures is calculated as follows:
  $$ Z-{score}_b=\frac{\left\langle {score}_{decoys}\right\rangle -{score}_{best\ decoy}}{\sigma_{decoys}} $$

where *score*_*best decoy*_scorenative is the score (energy) calculated for the best decoy structure, 〈*score*_*decoys*_〉 and *σ*_*decoys*_ are the average and standard deviation of score distributions of decoys proteins (the native and the best decoy are excluded), respectively.

6) F.E (20% Fraction Enrichment): which measures the fraction of the most accurate 20% decoys (the top 20% lowest RMSD structures) among the 20% best scoring decoys (by excluding the native structure) compared to that for the entire decoy set [19].

## Supplementary information

**Additional file 1.** The supplementary material includes additional figures and tables.

## Data Availability

The final list of PDB and chain IDs are provided in Additional file 1: Table S1. The CASP10–13 decoy set is publicly available at http://qbp.hzau.edu.cn/ANDIS/ and the 3DRobot decoy set is publicly available at http://zhanglab.ccmb.med.umich.edu/3DRobot/decoys.

## Abbreviations

KBP: Knowledge-based potential
PCA: Principal component analysis
