# Supplementary material for: Identification of native protein structures captured by principal interactions
Source: BMC Bioinformatics. 2019 Nov 21;20:604. doi: 10.1186/s12859-019-3186-6 (PMC6873546; doi:10.1186/s12859-019-3186-6)
Supplement: Supplementary file 1 — Additional file 1. The supplementary material includes additional figures and tables. [file 12859_2019_3186_MOESM1_ESM.docx]

**Supplementary information**

**Image representation of the CTE matrix**

- In the figures 1 and 2 the x-axis represents 210 variables and y-axis represents 1000 randomly selected protein structures from train data set. Each element of CTE corresponds to a rectangular area in the image. The values of the elements of CTE are indices into the current color map that determine the color of each patch. As shown in the figures 1 and 2, the values of some variables or interactions shown as black points are different from others and are very low. The interactions indicated by arrows are principal interactions elicited using presented method.


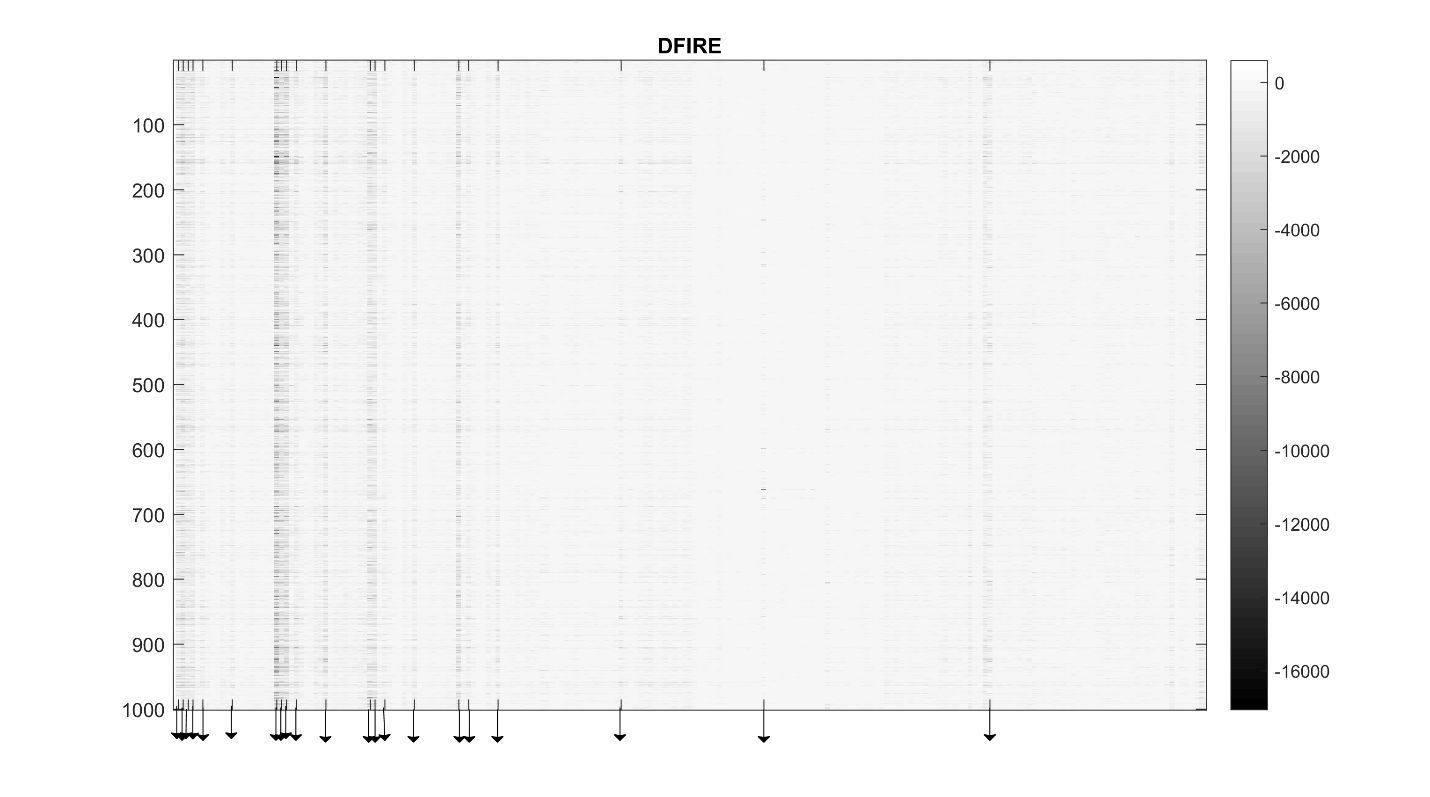


**Figure 1. Image representation of the CTE matrix for DFIRE.** (The principal interaction shown by arrows are from left to right: F-F, F-L, F-I, F-V, F-Y, F-A, L-L, L-I, L-V ,L-Y, L-A, I-I, I-V, I-Y, I-A, V-V, V-Y, V-A, Y-Y, C-C, and A-A, respectively)


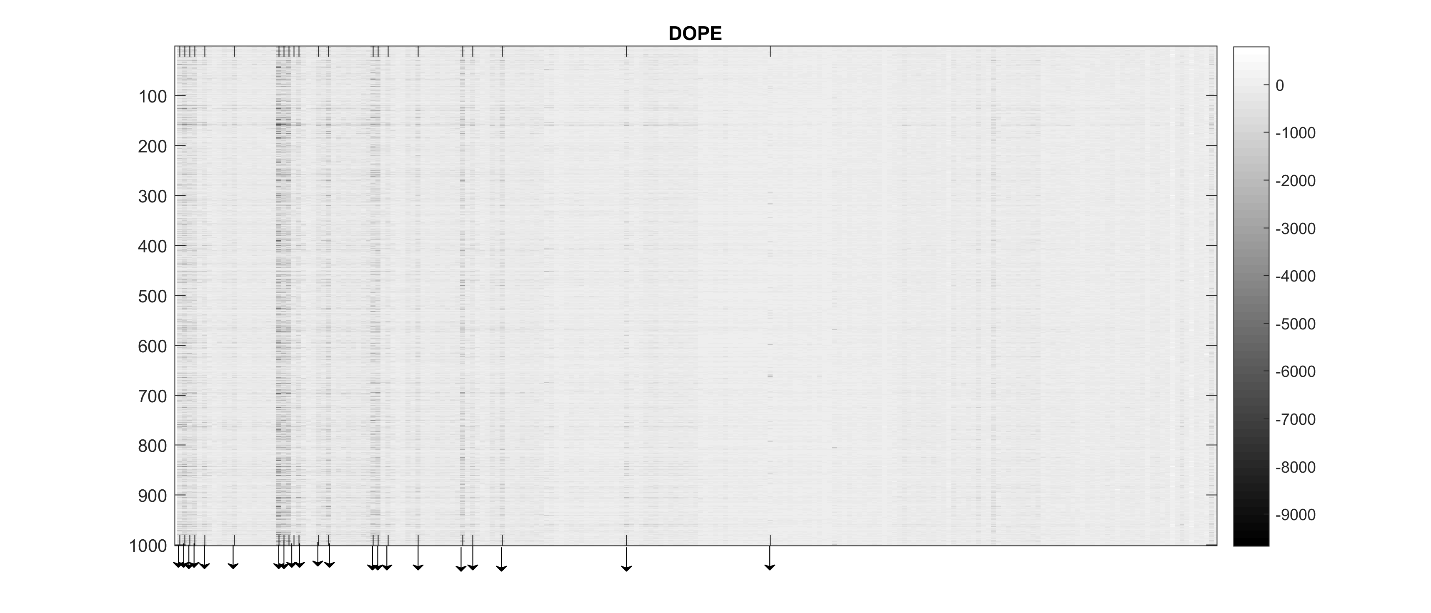


**Figure 2. Image representation of the CTE matrix for DOPE.** (The principal interaction shown by arrows are from left to right: F-F, F-L, F-I, F-V, F-Y, F-A, L-L, L-I, L-V ,L-W, L-Y, L-T, L-A, I-I, I-V, I-Y, I-A, V-V, V-Y, V-A, Y-Y, and C-C, respectively)

**Principal interactions:**

The contribution of interactions in three knowledge-based potential are shown in the following figures. The red dashed line represents the uniform contribution as discussed in method section.


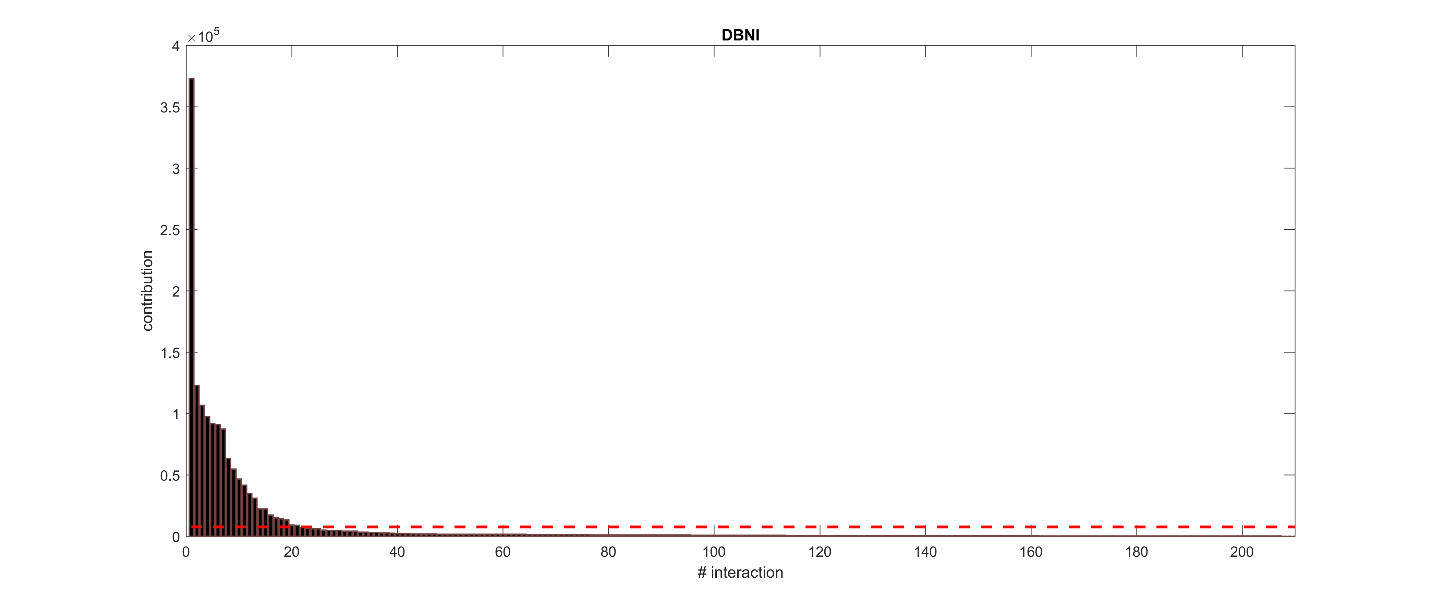
**Figure 3.** The contribution of interactions in DBNI.


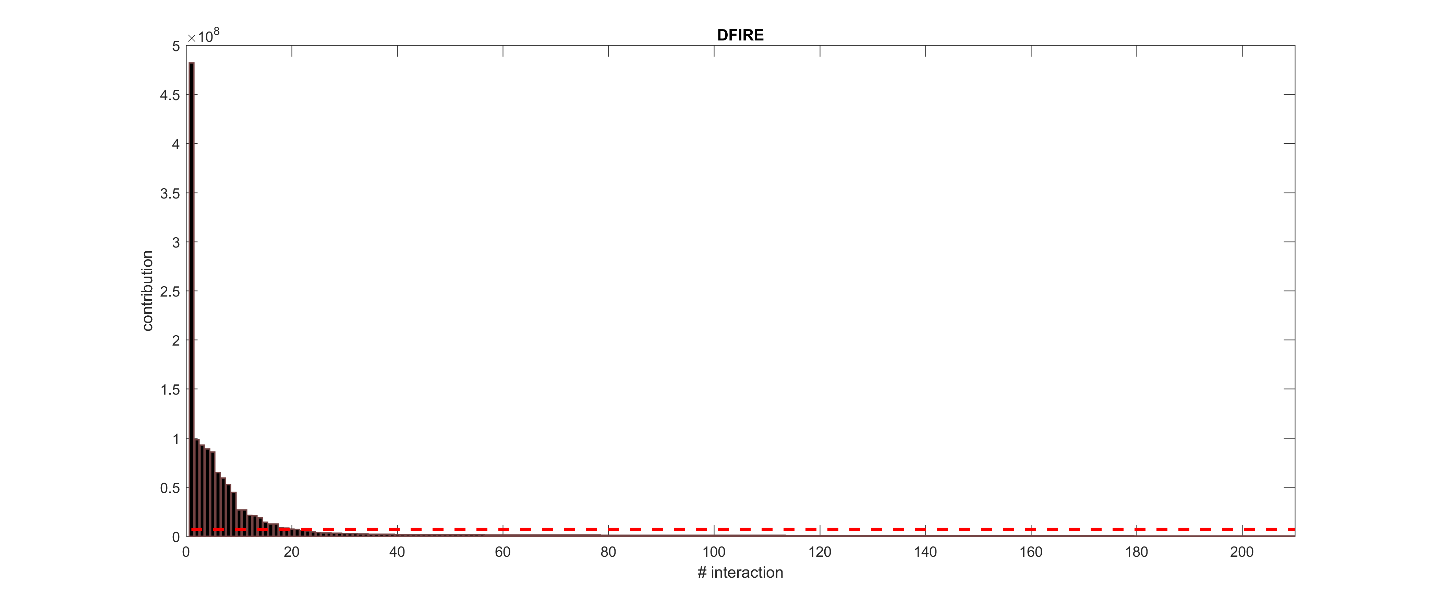


**Figure 4.** The contribution of interactions in DFIRE.


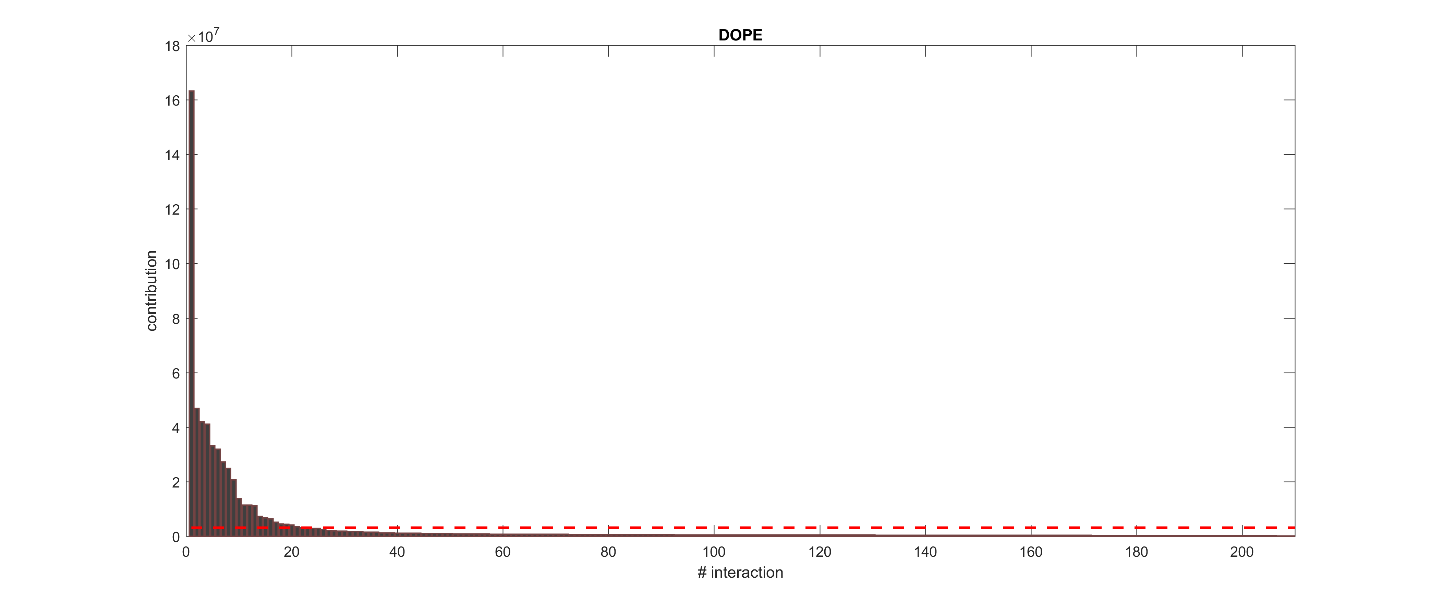


**Figure 5**.The contribution of interactions in DOPE.

**Table S1.** The list of PDB IDs used for train.

| 16PKA  1A2PA  1A4IA  1A62A  1A7SA  1A8DA  1ABAA  1AH7A  1AHOA  1AOEA  1ATGA  1B0BA  1B0UA  1B5EA  1B67A  1B6GA  1B8ZA  1B9OA  1BRTA  1BSMA  1BTEA  1BTKA  1BX7A  1C0PA  1C1DA  1C1KA  1C1LA  1C4QA  1C52A  1C75A  1C7KA  1CCWA  1CG5B  1CS1A  1CSEI  1CUOA  1CXCA  1CXQA  1D2SA  1D4OA  1D4TA  1D5TA  1D8WA  1DBFA  1DBWA  1DCIA  1DCSA  1DD9A  1DFMA  1DG6A  1DGFA  1DI6A  1DJ0A  1DK8A  1DL2A  1DLWA  1DP7P  1DS1A  1DYPA  1DYQA  1DYSA  1DZKA  1E19A  1E29A  1E2WA  1E4MM  1E58A  1E6UA  1E7LA  1E9GA  1EAJA  1EAQA  1EAZA  1EB6A  1EDGA  1EGWA  1EJ0A  1EJ8A  1EKQA  1ELKA  1ELUA  1EN2A  1EP0A  1ES5A  1ES9A  1ET1A  1EU1A  1EUVA  1EUWA  1EVLA  1EW0A  1EZGA  1F0LA  1F1EA  1F2TA  1F46A  1F74A  1F7DB  1F7LA  1F94A | 1F9VA  1FCQA  1FCYA  1FD3A  1FG7A  1FGYA  1FIUA  1FJ2A  1FL0A  1FM0D  1FO8A  1FP2A  1FR3A  1FSGA  1FT5A  1FVGA  1FYEA  1G12A  1G1TA  1G2QA  1G2RA  1G2YA  1G3PA  1G4IA  1G57A  1G5AA  1G61A  1G66A  1G6GA  1G6HA  1G6XA  1GA6A  1GCIA  1GEEA  1GHEA  1GJ7A  1GK7A  1GK8I  1GK9A  1GKMA  1GKPF  1GMUA  1GMXA  1GNLA  1GOIA  1GPPA  1GQIA  1GS5A  1GTVA  1GTZA  1GU2B  1GUTA  1GUUA  1GV9A  1GVDA  1GVEB  1GVFA  1GVZA  1GWEA  1GWMA  1GWUA  1GXMA  1GXUA  1GY6A  1GY7A  1GYOA  1GYXA  1GZ2A  1GZCA  1H12A  1H16A  1H1NB  1H2CA  1H32A  1H4AX  1H4GA  1H4XA  1H5QA  1H80A  1H97B  1H99A  1HDHB  1HDOA  1HFEL  1HLQA  1HNJA  1HQ1A  1HQKA  1HQSA  1HT6A  1HW1A  1HXHA  1HXIA  1HYOA  1HZ4A  1HZTA  1I0RA  1I0VA  1I12A  1I1NA | 1I1WA  1I24A  1I27A  1I2TA  1I4UA  1I52A  1I58A  1I60A  1I71A  1I8OA  1ID0A  1IDPA  1IFCA  1IFRA  1II5A  1IJQA  1IJYA  1IKPA  1IN4A  1INLA  1IO0A  1IO7A  1IOMA  1IOOA  1IQ6A  1IQQA  1IQZA  1IRQA  1IS3A  1ISUA  1IT2A  1ITXA  1IU8A  1IUJA  1IUQA  1IV3A  1IX9A  1IXHA  1IY8A  1IYBA  1IYNA  1J0PA  1J2JB  1J2RA  1J34A  1J3AA  1J3WA  1J77A  1J8UA  1J98A  1JA9A  1JATB  1JB3A  1JBEA  1JCDA  1JE0A  1JEKA  1JERA  1JETA  1JF3A  1JF8A  1JFBA  1JFUA  1JG1A  1JHJA  1JI7A  1JIGA  1JKEA  1JKXA  1JL0A  1JL1A  1JLJA  1JM1A  1JNDA  1JNIA  1JNRA  1JO0B  1JO8A  1JOVA  1JR8A  1JTVA  1JU2A  1JUHA  1JX6A  1JY2N  1JYKA  1JZ8A  1K2AA  1K38A  1K3IA  1K3XA  1K4IA  1K4NA  1K5CA  1K5NA  1K7CA  1K7JA  1K7KA  1K8UA  1KA1A | 1KAFA  1KB0A  1KDGA  1KGDA  1KGSA  1KJQA  1KKOA  1KMVA  1KNGA  1KNMA  1KOEA  1KQ1A  1KQ3A  1KQ6A  1KQFA  1KQPA  1KR4A  1KRHA  1KT6A  1KTHA  1KW3B  1KWFA  1KWGA  1KYFA  1L3KA  1L6RA  1L7AA  1L7MA  1L8NA  1L9LA  1L9XA  1LAMA  1LC0A  1LC5A  1LF7A  1LKKA  1LLFA  1LLNA  1LMIA  1LO6A  1LO7A  1LQ9A  1LQTA  1LQVA  1LR7A  1LS1A  1LU0A  1LU4A  1LUCA  1LV7A  1LYQA  1LZLA  1M15A  1M1FA  1M1QA  1M22A  1M2DA  1M2KA  1M2XA  1M40A  1M4IA  1M4JA  1M4LA  1M55A  1M65A  1M70A  1M9ZA  1MB3A  1MC2A  1MG7A  1MJ4A  1MJ5A  1MJNA  1MK0A  1MKKA  1MKZB  1MN8A  1MNNA  1MOQA  1MS9A  1MTPA  1MUWA  1MV8A  1MVOA  1MWQB  1MXGA  1MXRA  1N08A  1N13A  1N3LA  1N40A  1N45A  1N57A  1N62A  1N7OA  1N7SA  1NA3A  1NBUA  1NC5A  1NC7A | 1NEYA  1NF9A  1NFPA  1NKDA  1NKGA  1NLQA  1NM8A  1NN5A  1NNFA  1NNLA  1NNXA  1NOAA  1NOGA  1NOXA  1NPIA  1NQ7A  1NQJA  1NTHA  1NTVA  1NU0A  1NUYA  1NWWA  1NXCA  1NXMA  1NYCA  1NYKA  1NYTA  1NZ0A  1NZIA  1NZJA  1O04A  1O06A  1O1ZA  1O2DA  1O4RA  1O4YA  1O5XA  1O6VA  1O7JA  1O7QA  1O8VA  1O8XA  1O97C  1O98A  1O9GA  1O9IA  1OA2A  1OAAA  1OAIA  1OAQL  1OC2A  1OC7A  1OCYA  1OD3A  1OD6A  1ODMA  1ODZA  1OEWA  1OF8B  1OFWA  1OH4A  1OHLA  1OHPA  1OI0A  1OI6A  1OI7A  1OJJA  1OK0A  1OKIA  1OLRA  1OOHA  1OOTA  1OQJA  1OQVA  1ORRA  1OU8A  1OUWA  1OW4A  1OX0A  1OXXK  1OYGA  1OZ2A  1OZNA  1P0HA  1P0ZA  1P1MA  1P1XA  1P3CA  1P4CA  1P4OA  1P5DX  1P5ZB  1P6OA  1P9GA  1P9HA  1P9IA  1PB7A  1PBJA  1PE9A  1PKHA | 1PKOA  1PL4A  1PM1X  1PMHX  1PO5A  1PP0A  1PQ7A  1PTFA  1PVMA  1PWAA  1PWBA  1PZ4A  1PZ7A  1PZGA  1Q0RA  1Q1AA  1Q1FA  1Q35A  1Q4UA  1Q6OB  1Q6ZA  1Q7EA  1Q7LA  1QAUA  1QDDA  1QE3A  1QFTA  1QG8A  1QGVA  1QH4D  1QH5A  1QHQA  1QKSA  1QLWA  1QNRA  1QOWD  1QPCA  1QQ5A  1QQFA  1QREA  1QS1A  1QTWA  1QU9A  1QV1A  1QV9A  1QVEA  1QW2A  1QW9A  1QWGA  1QWKA  1QWOA  1QWYA  1QX2A  1QXYA  1R0MA  1R0RI  1R26A  1R2QA  1R45A  1R55A  1R5LA  1R5MA  1R62A  1R6DA  1R6XA  1R7JA  1R85A  1R9LA  1RA0A  1RCQA  1RDQI  1RFYA  1RG8B  1RH9A  1RIEA  1RJUV  1RK6A  1RKIA  1RKQA  1RKUA  1RL0A  1RM6A  1ROCA  1RP0A  1RROA  1RTQA  1RTTA  1RU4A  1RUTX  1RV9A  1RW1A  1RWHA  1RY6A  1RYLA  1RYOA  1RYQA  1S1DA  1S1FA  1S29A  1S2OA | 1S3CA  1S8NA  1S9RA  1S9UA  1SBYA  1SENA  1SFSA  1SFXA  1SG4A  1SH8A  1SHUX  1SJ1A  1SJWA  1SJYA  1SK7A  1SMOA  1SN9A  1SNNA  1SO7A  1SQSA  1SVFA  1SVSA  1SX5A  1SXRA  1SXVA  1SZ7A  1SZHA  1SZNA  1T1GA  1T1UA  1T1VA  1T3YA  1T61A  1T6CA  1T6FA  1T7FB  1T7MB  1T7RA  1T8KA  1T92A  1T9HA  1T9IA  1TBFA  1TC1A  1TG0A  1TGXA  1THMA  1THXA  1TJOA  1TKEA  1TKJA  1TKSA  1TO2I  1TP6A  1TQGA  1TQJA  1TT8A  1TU7A  1TUAA  1TUKA  1TVGA  1TVNA  1TYJA  1TZPA  1TZVA  1U07A  1U0KA  1U2HA  1U53A  1U69A  1U7GA  1U7IA  1U84A  1U8VC  1U9CA  1UAIA  1UASA  1UB3A  1UCDA  1UCRA  1UCSA  1UF5A  1UFOA  1UFYA  1UG6A  1UGIA  1UGXB  1UHEA  1UI0A  1UJPA  1UK8A  1UKKA  1UKUA  1UNQA  1UOYA  1UOZA  1UP9A  1UPQA  1UR1A  1URRA | 1URSA  1US0A  1US5A  1USCA  1USEA  1USGA  1USMA  1UTIA  1UUQA  1UUYA  1UWCA  1UWKA  1UWWA  1UXXX  1UXZA  1UZ3A  1UZKA  1V05A  1V0LA  1V0WA  1V2XA  1V30A  1V37A  1V3WA  1V5DA  1V5IB  1V5VA  1V6PA  1V6SA  1V70A  1V7RA  1V7WA  1V7ZA  1V8CA  1V8HA  1V9YA  1VBWA  1VC3B  1VD6A  1VDWA  1VE1A  1VE4A  1VEFA  1VF8A  1VFYA  1VH5A  1VHNA  1VHTA  1VHUA  1VIAA  1VIMA  1VIOA  1VJKA  1VJUA  1VK1A  1VKEA  1VKIA  1VKKA  1VL1A  1VL7A  1VLYA  1VMFA  1VMGA  1VMHA  1VMJA  1VP8A  1VQSA  1VR7A  1VRMA  1VRZA  1VYIA  1VYKA  1VYRA  1VZIA  1VZMA  1W0HA  1W0PA  1W1HA  1W23A  1W2IA  1W2LA  1W32A  1W4SA  1W53A  1W5QA  1W5RA  1W66A  1W6SA  1W70A  1W7BA  1W7CA  1W9SA  1WB4A  1WC2A  1WCGA  1WCUA  1WCV1  1WCWA  1WDDA  1WERA | 1WFBA  1WHIA  1WHZA  1WKQA  1WKRA  1WL4A  1WL8A  1WLUA  1WLYA  1WLZA  1WM3A  1WMAA  1WMDA  1WMHA  1WMSA  1WMWA  1WNAA  1WNYA  1WPAA  1WPNA  1WQJB  1WR8A  1WRIA  1WS8A  1WT6A  1WTJA  1WU4A  1WU9A  1WVFA  1WVHA  1WVQA  1WWIA  1WY3A  1WYXA  1WZAA  1WZDA  1X0TA  1X2IA  1X3SA  1X46A  1X54A  1X6IB  1X6OA  1X6ZA  1X7DA  1X8QA  1X91A  1X9DA  1X9IA  1XBIA  1XD3A  1XDNA  1XDZA  1XEOA  1XG0A  1XG4A  1XG5A  1XGKA  1XJUA  1XK7A  1XKNA  1XLQA  1XMKA  1XMTA  1XOCA  1XODA  1XPPA  1XQOA  1XRKA  1XS0A  1XSQA  1XSZA  1XTAA  1XTEA  1XU9A  1XUBA  1XVXA  1XYZA  1Y07A  1Y0HA  1Y0MA  1Y0PA  1Y0UA  1Y0YA  1Y1PA  1Y2KA  1Y2MA  1Y43A  1Y4WA  1Y55X  1Y5HA  1Y6XA  1Y7LA  1Y8AA  1Y93A  1Y9LA  1Y9UA  1Y9ZA  1YB3A  1YBKA | 1YC5A  1YD0A  1YD9A  1YE8A  1YFQA  1YG9A  1YLXA  1YMTA  1YN9A  1YNPA  1YPHC  1YPYA  1YQSA  1YS1X  1YS7A  1YT3A  1YU0A  1YUZA  1YW5A  1YXYA  1YYAA  1Z0JA  1Z0NA  1Z0WA  1Z1SA  1Z2NX  1Z2UA  1Z3EA  1Z3XA  1Z41A  1Z67A  1Z6MA  1Z6NA  1Z70X  1Z72A  1Z8GA  1Z9TA  1ZCEA  1ZD8A  1ZDYA  1ZGKA  1ZHVA  1ZHXA  1ZJAA  1ZK4A  1ZK5A  1ZKEA  1ZKPA  1ZL0A  1ZLBA  1ZLMA  1ZMAA  1ZMIA  1ZMMA  1ZO2A  1ZOIA  1ZR6A  1ZUUA  1ZUYA  1ZV1B  1ZVAA  1ZX6A  1ZZKA  1ZZWA  2A0BA  2A26A  2A28A  2A35A  2A3NA  2A4XA  2A5JA  2A6VA  2A6ZA  2A8NA  2A8YA  2ABSA  2AEBA  2AEXA  2AGKA  2AH6A  2AHFA  2AHNA  2AIBA  2AKFA  2AKZA  2AMLA  2ANXA  2AP3A  2APJA  2AQPA  2ARCA  2ARRA  2ASBA  2ASFA  2ASKA  2AU7A  2AXWA  2AYDA  2AYHA  2B06A | 2B0AA  2B0VA  2B2HA  2B3FA  2B3GB  2B3HA  2B4HA  2B5AA  2B5WA  2B69A  2B7UA  2B82A  2B97A  2B9DA  2BAYA  2BBRA  2BCEA  2BCGY  2BCMB  2BDRA  2BF6A  2BF9A  2BFDA  2BGKA  2BH4X  2BHUA  2BJDA  2BJIA  2BJKA  2BK9A  2BKFA  2BKLA  2BKMA  2BKXA  2BL8A  2BLNA  2BMOA  2BO9B  2BOGX  2BPDA  2BRFA  2BRYA  2BSYA  2BT6A  2BU3A  2BV2A  2BW8A  2BWFA  2BWQA  2BWRA  2BZ1A  2BZVA  2C07A  2C0AA  2C0CA  2C0HA  2C0ZA  2C1IA  2C1VA  2C2NA  2C3NA  2C3VA  2C46A  2C4WA  2C5AA  2C60A  2C61A  2C6UA  2C71A  2C78A  2C8SA  2C92A  2C9JA  2CALA  2CARA  2CB8A  2CBZA  2CC0A  2CC6A  2CCQA  2CDCA  2CDPA  2CE0A  2CF7E  2CG7A  2CI1A  2CIAA  2CIBA  2CIOA  2CIRA  2CIUA  2CIWA  2CJLA  2CJTA  2CKKA  2CKSA  2CM2A  2CNQA  2CPGA  2CS7A | 2CUAB  2CVEA  2CVIA  2CWSA  2CXAA  2CXNA  2CXYA  2CYJA  2CZ2A  2CZDA  2CZLA  2CZQA  2CZSA  2D1SA  2D3DA  2D3MA  2D3YA  2D5MA  2D5WA  2D68A  2D8DA  2DDRA  2DDXA  2DE3A  2DEBA  2DEJA  2DG5A  2DHOA  2DJIA  2DKJA  2DKOA  2DLBA  2DPFA  2DPLA  2DRIA  2DRMA  2DS5A  2DSKA  2DT4A  2DT8A  2DTJA  2DTXA  2DVMA  2DWUA  2DXAA  2DXUA  2DY0A  2DY1A  2E0QA  2E2RA  2E3HA  2E3NA  2E3ZA  2E4TA  2E5FA  2E6FA  2E7ZA  2E9LA  2EABA  2EAQA  2EB4A  2EBBA  2ECUA  2EGVA  2EH3A  2EHGA  2EHPA  2EHZA  2EIXA  2EIYA  2EKPA  2ELCA  2ENDA  2EPLX  2EQ6A  2ERFA  2ERWA  2ET1A  2EV1A  2EVEA  2EW0A  2EWHA  2EX2A  2F01A  2F1KA  2F22A  2F23A  2F3YB  2F46A  2F5TX  2F60K  2F62A  2F69A  2F6UA  2F8AA  2F8YA  2F91A  2F9HA  2F9NA  2FAOA | 2FB6A  2FBAA  2FCLA  2FCOA  2FCTA  2FCWA  2FE5A  2FFYA  2FG1A  2FGOA  2FH1A  2FHPA  2FI1A  2FJ8A  2FKKA  2FL4A  2FLHA  2FMAA  2FN9A  2FNUA  2FP1A  2FR2A  2FR5A  2FRGP  2FSQA  2FSRA  2FSTX  2FTRA  2FUKA  2FULA  2FUPA  2FVYA  2FWHA  2FYFA  2G1UA  2G29A  2G2CA  2G30A  2G3RA  2G5RA  2G6FX  2G6YA  2G7BA  2G7OA  2G7SA  2G81I  2G84A  2G8SA  2GASA  2GB4B  2GCUA  2GECA  2GF3A  2GGCA  2GHSA  2GJ3A  2GJ4A  2GKEA  2GKGA  2GKPA  2GL5A  2GLZA  2GMWA  2GMYA  2GOMA  2GPIA  2GQ1A  2GQTA  2GQWA  2GRRB  2GS5A  2GS8A  2GSOA  2GU9A  2GUDA  2GUHA  2GUIA  2GVKA  2GW3A  2GWMA  2GXGA  2GXQA  2GYQA  2GZ4A  2GZQA  2GZSA  2GZVA  2H1VA  2H2BA  2H2TB  2H30A  2H3LA  2H4VA  2H5CA  2H6FA  2H7ZB  2H8EA  2H8GA  2H8OA  2HA8A | 2HALA  2HBAA  2HBTA  2HBWA  2HC1A  2HD9A  2HDOA  2HEUA  2HHCA  2HHGA  2HHJA  2HI0A  2HINA  2HIYA  2HLRA  2HLYA  2HO2A  2HOXA  2HP0A  2HPWA  2HQSH  2HQXA  2HSJA  2HTDA  2HUHA  2HW2A  2HWNE  2HX0A  2HX5A  2HXSA  2HYKA  2HZLA  2I0KA  2I24N  2I33A  2I3DA  2I49A  2I4AA  2I51A  2I53A  2I5FA  2I5UA  2I5VO  2I61A  2I8TA  2IA1A  2IA7A  2IAYA  2IBDA  2IBNA  2IBPA  2IC2A  2IC6A  2ICIA  2ICRA  2II2A  2IIMA  2IJ2A  2ILKA  2IMHA  2IMIA  2IMJA  2IMQX  2INWA  2IP6A  2IT2A  2IU5A  2IUMA  2IUWA  2IVYA  2IW1A  2IWAA  2IWNA  2IWRA  2IXMA  2IXTA  2IYVA  2IZ6A  2IZRA  2IZXA  2J05A  2J1AA  2J23A  2J2JA  2J43A  2J5GA  2J5YA  2J6LA  2J6VA  2J82A  2J8BA  2J8KA  2J8MA  2J8WA  2J9OC  2JAEB  2JBAA  2JC5A  2JC9A  2JCBA | 2JDAA  2JDCA  2JE6A  2JEKA  2JENA  2JEPA  2JFRA  2JG0A  2JHFB  2JIKA  2JILA  2JISA  2JJNA  2JJUA  2JKHA  2JKUA  2MCMA  2MHRA  2NLIA  2NLRA  2NLSA  2NLVA  2NMLA  2NN5A  2NNUA  2NPNA  2NQTA  2NQWA  2NR7A  2NRRA  2NUHA  2NW8A  2NWFA  2NWRA  2NXVA  2NXWA  2NYBA  2O0AA  2O0BA  2O0MA  2O1QA  2O23A  2O2KA  2O2XA  2O5GB  2O60B  2O6NA  2O6PA  2O6YA  2O7AA  2O7IA  2O7RA  2O8LA  2O8QA  2O9SA  2OA2A  2OAAA  2OB3A  2OB5A  2OC3A  2OCTA  2ODIA  2ODKA  2OFCA  2OFKA  2OFZA  2OG5A  2OGTA  2OH1A  2OIZA  2OKFA  2OKTA  2OLMA  2OLNA  2OMLA  2OOAA  2OOCA  2OPCA  2OPGA  2OPJA  2OPLA  2OQGA  2OQZA  2OR7A  2ORDA  2ORWA  2OS0A  2OS5A  2OSXA  2OU5A  2OUSA  2OV0A  2OVGA  2OXCA  2OXGY  2OY9A  2OYOA  2OZHA  2OZJA  2OZNA | 2OZTA  2P0NA  2P0SA  2P14A  2P17A  2P2RA  2P2SA  2P39A  2P4FA  2P4HX  2P51A  2P5KA  2P6WA  2P7OA  2P8IA  2P9WA  2PA7A  2PAGA  2PBDV  2PC1A  2PEBA  2PEFA  2PEZA  2PFIA  2PGEA  2PGNA  2PHNA  2PKFA  2PLRA  2PLXB  2PMKA  2PN6A  2PNDA  2PNEA  2POFA  2POSA  2PQ7A  2PQ8A  2PQCA  2PQXA  2PR5A  2PR7A  2PRVA  2PRXA  2PU3A  2PUYA  2PV2A  2PVBA  2PWAA  2PXXA  2PY5A  2PYQA  2PYXB  2Q0LA  2Q1SA  2Q2FA  2Q2IA  2Q3GA  2Q3WA  2Q52A  2Q5CA  2Q6KA  2Q7DA  2Q7WA  2Q8KA  2Q8XB  2Q9KA  2Q9OA  2QA9E  2QAPA  2QB7A  2QE8B  2QEDA  2QF4A  2QFAA  2QG1A  2QGUA  2QHLA  2QHSA  2QIKA  2QIPA  2QJWD  2QJZB  2QKVA  2QL8A  2QLTA  2QLWA  2QMLA  2QNGA  2QNKA  2QNLA  2QNTA  2QO4A  2QPXA  2QR6A  2QRLA  2QSBA  2QSKA  2QSWA  2QT1A |
| --- | --- | --- | --- | --- | --- | --- | --- | --- | --- | --- | --- | --- | --- | --- | --- |

**Table S1.** (Continued)

| 2QUDA  2QVGA  2QVKA  2QXFA  2QXIA  2QZCA  2R01A  2R0XA  2R16A  2R1JL  2R2ZA  2R31A  2R4IB  2R4QA  2R5OA  2R6JA  2R6UA  2R6VA  2R751  2R78A  2R8EA  2R8OA  2R8QA  2R9FA  2RA6A  2RA9A  2RAFB  2RB7A  2RB8A  2RBDB  2RBKA  2RC3A  2RC8A  2RDGA  2RDQA  2RE2A  2RFFA  2RFRA  2RFVA  2RH2A  2RHFA  2RHWA  2RI0A  2RI7A  2RIKA  2RILA  2RK3A  2RK5A  2RK9A  2RKLA  2RKNA  2RKQA  2RKVA  2RL8A  2TPSA  2UU8A  2UUQA  2UUYB  2UV4A  2UVKA  2UVOA  2UX9A  2UXWA  2UY2A  2UYTA  2V03A  2V1MA  2V1QB  2V25A  2V27A  2V2GA  2V2KA  2V33A  2V3GA  2V3IA  2V3ZA  2V4VA  2V4XA  2V52M  2V6KA  2V6UA  2V6VA  2V76A  2V7FA  2V89A  2V8FA  2V8IA  2V8TB  2V9BB  2V9LA  2V9VA  2VB1A  2VBFA  2VBKA  2VC8A  2VCHA  2VCLA  2VCNA  2VD8A  2VE8A | 2VEZA  2VFOA  2VFRA  2VH3A  2VHAA  2VIFA  2VIMA  2VK2A  2VK8A  2VLAA  2VMHA  2VN6A  2VNGA  2VOCA  2VOKA  2VOVA  2VPAA  2VPBB  2VPNA  2VPTA  2VQ2A  2VQ8A  2VQPA  2VQRA  2VS0A  2VTCA  2VUVA  2VVGA  2VW8A  2VWFA  2VWSA  2VXNA  2VXTI  2VY8A  2VYOA  2VZPA  2W15A  2W1JA  2W1RA  2W1SA  2W1VA  2W20A  2W2JA  2W31A  2W39A  2W3GA  2W3PA  2W3QA  2W3ZA  2W40A  2W47A  2W50A  2W5AA  2W5QA  2W6AA  2W72A  2W7AA  2W7ZA  2W87A  2W8TA  2W8XA  2W91A  2W9HA  2WAGA  2WAWA  2WB9A  2WBFX  2WBQA  2WCJA  2WCWA  2WDCA  2WE5B  2WFIA  2WFWA  2WH6A  2WH7A  2WHLA  2WI8A  2WIYA  2WJ5A  2WK1A  2WKJA  2WKKA  2WL1A  2WLRA  2WLTA  2WLVA  2WM5A  2WMFA  2WN3A  2WNFA  2WNKA  2WNVA  2WNXA  2WOLA  2WOYA  2WQ1A  2WQ4A  2WQFA  2WQKA | 2WRAA  2WRYA  2WSBA  2WSDA  2WTGA  2WTMA  2WTPA  2WU9B  2WUHA  2WUJA  2WVFA  2WW6A  2WWEA  2WWXB  2WY4A  2WYUA  2WZ8A  2WZ9A  2WZBA  2WZOA  2X1FA  2X2SA  2X32A  2X3HA  2X46A  2X49A  2X4KA  2X4LA  2X5NA  2X5OA  2X5PA  2X5XA  2X5YA  2X7KA  2X7MA  2X8SA  2X9GA  2X9OA  2X9ZA  2XC2A  2XDPA  2XDWA  2XEPA  2XETA  2XEUA  2XEVA  2XFDA  2XFRA  2XGUA  2XHFB  2XHGA  2XHIA  2XI8A  2XIOA  2XIRA  2XJ4A  2XJPA  2XKIA  2XMJA  2XN2A  2XN6A  2XNQA  2XOLA  2XOMA  2XPWA  2XR6A  2XRHA  2XRWA  2XRYA  2XS2A  2XTPA  2XTSA  2XTTA  2XUVA  2XVMA  2XW6A  2XW9A  2XWLA  2XWSA  2XWVA  2XZ2A  2XZIA  2Y0OA  2Y24A  2Y27A  2Y3CA  2Y53A  2Y5PD  2Y6XA  2Y71A  2Y78A  2Y7EA  2Y8EA  2Y8KA  2Y8YA  2Y9FA  2Y9UA  2YB6A  2YBYA  2YC3A | 2YCDA  2YD6A  2YEXA  2YFOA  2YH6A  2YHGA  2YIMA  2YK3A  2YLNA  2YMVA  2YN0A  2YNAA  2YOGA  2YV9A  2YVEA  2YVTA  2YWIA  2YWKA  2YWLA  2YXMA  2YXOA  2YYKA  2YZVA  2YZYA  2Z08A  2Z0JA  2Z26A  2Z3HA  2Z4UA  2Z51A  2Z5WA  2Z6OA  2Z6RA  2Z72A  2Z8XA  2Z98A  2ZA4B  2ZADA  2ZATA  2ZBLA  2ZBXA  2ZCMA  2ZCWA  2ZDPA  2ZEXA  2ZFDA  2ZFIA  2ZHJA  2ZHNA  2ZIBA  2ZK9X  2ZL7A  2ZO6A  2ZOUA  2ZPMA  2ZPOA  2ZPTX  2ZQ0A  2ZS0A  2ZSCA  2ZUXA  2ZW2A  2ZWSA  2ZXYA  2ZYOA  2ZZVA  3A02A  3A03A  3A07A  3A09A  3A0SA  3A0YA  3A16A  3A1SA  3A21A  3A2ZA  3A35A  3A3DA  3A4RA  3A57A  3A5FA  3A72A  3A7IA  3A7LA  3A8GA  3A99A  3A9BA  3A9FA  3A9JC  3A9SA  3A9ZA  3AALA  3AAMA  3ACHA  3ACXA  3AGNA  3AGTA  3AJ3A  3AJ4A  3AJ6A | 3AJ7A  3AJDA  3AJXA  3AK2A  3AK8A  3AKBA  3AKEA  3AKSA  3ALFA  3ALJA  3AMNA  3AMRA  3AOFA  3AOWA  3ARXA  3ASLA  3AYJA  3AZDA  3B0FA  3B0GA  3B0TA  3B0XA  3B12A  3B1NA  3B34A  3B4NA  3B4QA  3B4UA  3B5MA  3B5NA  3B5OA  3B64A  3B79A  3B9TB  3B9WA  3BA1A  3BA3A  3BB0A  3BB7A  3BC9A  3BCWB  3BD1A  3BDIA  3BE4A  3BEDA  3BERA  3BEUA  3BEXA  3BF7A  3BFOA  3BGUA  3BH4A  3BHDA  3BHQB  3BHWA  3BJEA  3BKRA  3BKWB  3BLNA  3BM7A  3BMVA  3BMXA  3BMZA  3BNJA  3BO5A  3BO6A  3BOEA  3BONA  3BPKA  3BPTA  3BPUA  3BPVA  3BQPA  3BQXA  3BR8A  3BRCA  3BS2A  3BS4A  3BT5A  3BUUA  3BUXB  3BVFA  3BVXA  3BWDD  3BWHA  3BWVB  3BWXA  3BWZA  3BY4A  3BZYB  3C2UA  3C3YA  3C5EA  3C6AA  3C70A  3C7FA  3C7MA  3C8CA  3C8LB  3C8WC | 3C8YA  3C8ZA  3C9AA  3C9UA  3C9ZA  3CA7A  3CAOA  3CAYA  3CB0A  3CBWA  3CBZA  3CCGA  3CECA  3CG1A  3CH0A  3CHJA  3CHMA  3CHVA  3CI6A  3CIJA  3CJMA  3CJSA  3CJWA  3CKCA  3CKKA  3CKMA  3CL6A  3CLMA  3CM3A  3CMBD  3COVA  3CP5A  3CP7A  3CQLA  3CRNA  3CSEA  3CT5A  3CT6A  3CTPA  3CTZA  3CU4A  3CU9A  3CUZA  3CVBA  3CWNA  3CWRB  3CX2A  3CXMA  3CXNA  3CYPB  3CZ1A  3CZXA  3D02A  3D06A  3D0JA  3D1KA  3D1PA  3D22A  3D2QA  3D40A  3D4EA  3D59A  3D5PA  3D7JA  3D9NA  3D9XA  3DA8B  3DAQA  3DASA  3DB7A  3DEOA  3DFFA  3DFGA  3DG6A  3DGTA  3DHAA  3DI4B  3DJEA  3DK9A  3DKMA  3DKRA  3DLCA  3DMGA  3DMOA  3DNJA  3DO8A  3DOUA  3DQPA  3DQYA  3DR4A  3DRFA  3DS4A  3DSBA  3DSKA  3DSOA  3DUWA  3DWGA  3DWVA  3DXLA  3DXYA | 3DY0A  3E0EA  3E0XA  3E10B  3E13X  3E23A  3E2DA  3E2OA  3E2VA  3E3MA  3E3UA  3E48A  3E4GA  3E4VA  3E5TA  3E7RL  3E8MD  3E8OB  3E8TA  3E8YX  3E9TA  3EA6A  3EAZA  3EBTA  3EBVA  3EC6A  3EC9A  3EDHA  3EDNA  3EDOB  3EERA  3EF4A  3EF8A  3EJ9A  3EJFA  3EJVA  3EKGA  3EKIA  3ELFA  3ELXA  3EN0A  3EO6B  3EOIA  3EOJA  3EPRA  3EQXB  3ER7A  3ERPA  3ESSA  3ETJA  3EURA  3EVOA  3EW1A  3EWYA  3EXVA  3EY6A  3EYEA  3EYIA  3F0DA  3F14A  3F1LA  3F1PB  3F2ZA  3F40A  3F43A  3F44A  3F4SA  3F5LA  3F5VA  3F6CA  3F6VA  3F6YA  3F7EA  3F7XA  3F8DA  3F8XD  3F9MA  3F9XA  3FBGA  3FCNA  3FDEA  3FDXA  3FEDA  3FEGA  3FG9A  3FGHA  3FGVA  3FGYA  3FH1A  3FIAA  3FILA  3FIQA  3FJMA  3FJUB  3FK8A  3FMYA  3FO3A  3FOJA  3FPCA  3FPWA | 3FR7A  3FRHA  3FSAA  3FSOA  3FSSA  3FTDA  3FUCA  3FUTA  3FW9A  3FWKA  3FXAA  3FYNA  3FZ4A  3G02A  3G0KA  3G16A  3G1PA  3G21A  3G36A  3G46A  3G48A  3G4EA  3G5SA  3G5TA  3G7NA  3G7RA  3G89A  3G91A  3G9XA  3G9YA  3GA3A  3GA4A  3GA7A  3GAEA  3GBWA  3GC6A  3GD6A  3GDMA  3GE3A  3GFAA  3GG7A  3GHAA  3GHJA  3GIPA  3GIRA  3GIUA  3GIWA  3GJ0A  3GJUA  3GJYA  3GKJA  3GKRA  3GMGA  3GMOA  3GMXB  3GNEA  3GNLA  3GNZP  3GO5A  3GOCB  3GOEA  3GOHA  3GOXA  3GP2B  3GP6A  3GPIA  3GPKA  3GR3A  3GRDB  3GRUA  3GV3A  3GVEA  3GWAA  3GWIA  3GWKC  3GXHA  3GY9A  3GYBA  3GYLB  3GZAB  3GZBF  3GZGA  3GZRB  3GZXA  3H0NA  3H0OA  3H0UA  3H31A  3H3HA  3H3LB  3H4NA  3H4OA  3H4TA  3H4XA  3H5JA  3H6JA  3H74A  3H75A  3H79A  3H7HA | 3H7IA  3H7UA  3H8GF  3H9CA  3H9MA  3HCNA  3HDXA  3HF5A  3HFOA  3HH7A  3HHIA  3HHPA  3HHTA  3HHYA  3HIDA  3HISA  3HJ4A  3HKWA  3HLXA  3HLZA  3HM4B  3HMCA  3HMZA  3HNXA  3HO6A  3HO7A  3HOIA  3HP4A  3HP7A  3HPCX  3HPWC  3HR6A  3HS3A  3HT1A  3HTNA  3HU5A  3HUHA  3HUPA  3HV2A  3HV8A  3HWUA  3HX8B  3HYGA  3HYNA  3HZ8A  3HZAA  3HZPA  3I06A  3I10A  3I24A  3I2KA  3I2VA  3I2ZB  3I33A  3I3FA  3I45A  3I47A  3I4GA  3I4OA  3I6CA  3I7MA  3I94A  3IARA  3IB5A  3IB7A  3IE7A  3IEZA  3IFEA  3IFNP  3IGSA  3IHSA  3IISM  3IJLA  3IKWA  3IM9A  3IMKA  3IP0A  3IPCA  3IPNA  3IQLA  3IQTA  3IQUA  3IR4A  3IRPX  3IRVA  3IS3A  3ISXA  3IT3A  3ITFA  3ITQA  3IUOA  3IUWB  3IV0A  3IV4A  3IVYA  3IVZA  3IWFA  3IWLA  3IXLA  3JQ0A | 3JQ1A  3JRVA  3JS8A  3JSCA  3JSYA  3JTMA  3JTZA  3JU0A  3JU4A  3JUDA  3JUMA  3JXOA  3JYOA  3JYZA  3JZYA  3K01A  3K05A  3K0BA  3K12A  3K1UA  3K1WA  3K1ZA  3K21A  3K2ZA  3K5JA  3K6IA  3K6MA  3K6YA  3K7IB  3K7PA  3K89A  3K9WA  3KANA  3KB5A  3KB9A  3KC2A  3KE7B  3KEOA  3KEVA  3KFAA  3KFFA  3KGKA  3KGYB  3KH1B  3KHFA  3KIZA  3KKFA  3KKGA  3KM5A  3KMAA  3KMHA  3KNBA  3KORA  3KPBA  3KPEA  3KRSA  3KTCB  3KTPB  3KTZA  3KU3B  3KUVA  3KWEA  3KWKA  3KWRA  3KXQA  3KYJA  3KYZA  3KZ5E  3KZDA  3L0FA  3L12A  3L1EA  3L1NA  3L1WA  3L32A  3L3UA  3L41A  3L46A  3L4AA  3L4EA  3L4NA  3L4RA  3L51A  3L5LA  3L6BA  3L77A  3L81A  3L84A  3L8AA  3L8QA  3L8WA  3L9CA  3LAAA  3LAEA  3LAGA  3LASA  3LAXA  3LD7A  3LE1A  3LEDA | 3LF5A  3LFJA  3LFKA  3LFRA  3LG3A  3LHCA  3LHIA  3LHNA  3LHQA  3LHSA  3LJKA  3LJMA  3LJWA  3LK7A  3LKMA  3LLOA  3LLUA  3LLXA  3LM3A  3LMZA  3LNYA  3LO8A  3LOPA  3LQ0A  3LQBA  3LQWA  3LRTA  3LS9A  3LSNA  3LT7A  3LTIA  3LW3A  3LWCA  3LWXA  3LX3A  3LY0A  3LYDA  3LYEA  3LYHB  3LYPA  3M07A  3M0FA  3M0MA  3M0ZA  3M1XA  3M3GA  3M3PA  3M5QA  3M66A  3M6WA  3M6ZA  3M73A  3M7AB  3M8JA  3M97X  3M9LA  3MABA  3MAOA  3MB5A  3MBKA  3MBRX  3MC3A  3MCWB  3MCXA  3MD1A  3MD7A  3MD9A  3MDMA  3MDQA  3MDUA  3MDXA  3ME7A  3MEAA  3MILA  3MJEA  3MJFA  3ML1A  3MN5S  3MNGA  3MOYA  3MOZA  3MPCA  3MQDA  3MQHA  3MQZA  3MR0A  3MSTA  3MT0A  3MU7A  3MVCA  3MVGA  3MVSA  3MWXA  3MWZA  3MXNA  3MXZA  3MYBA  3MYXA  3MZ0A  3MZ2A | 3MZFA  3N08A  3N0RA  3N0UA  3N0XA  3N10A  3N17A  3N1FC  3N1SA  3N2WA  3N3MA  3N4IB  3N4JA  3N5AA  3N6YA  3N6ZA  3N79A  3NBKA  3NBMA  3NCLA  3NCOA  3ND1A  3NDDA  3NDHA  3NDOA  3NE8A  3NEDA  3NEPX  3NEQA  3NEUA  3NFKA  3NFTA  3NFWA  3NGGA  3NGPA  3NHIA  3NIRA  3NIYA  3NJ2A  3NKEA  3NNBA  3NO0A  3NO2A  3NO7A  3NOHA  3NPDA  3NPKA  3NR5A  3NRED  3NRFB  3NS6A  3NSOA  3NTVA  3NUAA  3NUFA  3NV1A  3NVSA  3NVWA  3NWPA  3NWRA  3NYCA  3NYQA  3NYTA  3NYYA  3NZBX  3NZLA  3NZNA  3O12A  3O1CA  3O22A  3O2RA  3O2TA  3O3YA  3O46A  3O4PA  3O5QA  3O8MA  3O8QA  3O94A  3O9ZA  3OA2A  3OA3A  3OAJA  3OBLA  3OBQA  3OC7A  3OCJA  3OCUA  3OD5A  3ODTA  3ODVA  3OE3A  3OF5A  3OFGA  3OG2A  3OHEA  3OIGA  3OJ7A  3OLLA  3OM0A | 3OMDA  3OMYA  3ON9A  3ONDA  3OO8A  3OOOA  3OOUA  3OOXA  3OP4A  3OQPA  3ORKA  3ORUA  3OT1A  3OTIA  3OTXA  3OU2A  3OUFA  3OUGA  3OV5A  3OV9A  3OX7P  3OXPA  3OYVA  3OZ2A  3OZYA  3P02A  3P0BA  3P0FA  3P0KA  3P1GA  3P2CA  3P3OA  3P4HA  3P6IA  3P73A  3PA6A  3PC3A  3PD7A  3PE6A  3PE8A  3PESA  3PEYA  3PF6A  3PFBA  3PFEA  3PFGA  3PHHA  3PIDA  3PIUA  3PJPA  3PJYA  3PKAA  3PKVA  3PL8A  3PLUA  3PMEA  3PMOA  3PMSA  3PN3B  3PNAA  3PNZA  3PO8A  3PODA  3POHA  3POJA  3PONA  3POWA  3PP2A  3PP4P  3PP5A  3PP9A  3PPLA  3PPTA  3PQAA  3PQHA  3PSMA  3PT5A  3PU9A  3PVEA  3PVHA  3PVIA  3PX8X  3PZGA  3Q12A  3Q1CA  3Q1XA  3Q2BA  3Q2IA  3Q46A  3Q4OA  3Q64A  3Q6BA  3Q7RA  3Q8JA  3QATA  3QB8A  3QC0A  3QC7A  3QFTA  3QGUA | 3QHBA  3QHPA  3QIOA  3QJAA  3QK8A  3QL9A  3QNSA  3QOOA  3QP4A  3QPAA  3QR7A  3QSDA  3QU3A  3QU5A  3QVPA  3QWBA  3QX1A  3QXCA  3QXZA  3QY1A  3QZBA  3QZMA  3QZRA  3R0NA  3R0VA  3R2KA  3R2QA  3R3QA  3R3RA  3R3SA  3R41A  3R4ZA  3R5GA  3R5LA  3R5TA  3R62A  3R68A  3R6DA  3R87A  3R8JA  3R9FA  3RD5A  3RFEA  3RGAA  3RHGA  3RIQA  3RJTA  3RJUA  3RKGA  3RL5A  3RLGA  3RM3A  3RNJA  3RNQA  3RO0A  3RO3A  3RO8A  3ROBA  3ROFA  3RPCA  3RPDA  3RPEA  3RPZA  3RQ9A  3RQTA  3RR6A  3RRIA  3RT2A  3RTLA  3RWNA  3RX9A  3RY0A  3RY4A  3S0AA  3S2JA  3S2RA  3S44A  3S4EA  3S57A  3S5MA  3S69A  3S6EA  3S6FA  3S83A  3S8MA  3S8SA  3S9XA  3SBMA  3SC7X  3SCYA  3SD2A  3SEBA  3SEEA  3SFJA  3SG0A  3SGGA  3SGZA  3SH4A  3SHGA  3SIGA | 3SILA  3SJMA  3SK2A  3SK7A  3SKXA  3SLZA  3SMJA  3SMVA  3SNFA  3SNOA  3SOEA  3SOJA  3SOVA  3SP7A  3SQZA  3SRIB  3SS7X  3SU6A  3SUJA  3SUKA  3SUUA  3SWOA  3SX2A  3SXMA  3SXXA  3SZ3A  3SZAA  3SZHA  3SZVA  3SZYA  3T0OA  3T2CA  3T47A  3T49A  3T4LA  3T7HA  3T7LA  3T7VA  3T8JA  3T90A  3T92A  3T9WA  3TA6A  3TAKA  3TBNA  3TBOA  3TC2A  3TC3A  3TC8A  3TD3A  3TDNA  3TDUA  3TEWA  3TFJA  3TG0A  3TG2A  3TG7A  3TJ4A  3TJ8A  3TJLA  3TJMA  3TJRA  3TKFA  3TLOA  3TM8A  3TN4A  3TNLA  3TNYA  3TODA  3TOSA  3TPDA  3TQ2A  3TQ5A  3TQEA  3TQLA  3TRDA  3TS3A  3TT9A  3TTVA  3TU8A  3TUTA  3TVJA  3TVTA  3TWYA  3TY4A  3TYSA  3U23A  3U2UA  3U3GD  3U3LC  3U3ZA  3U5SA  3U62A  3U65B  3U6GA  3U6PA  3U7QA  3U7RA  3U80A  3U8EA | 3U8IA  3U97A  3U99A  3U9RB  3U9WA  3UB6A  3UBDA  3UC7A  3UE2A  3UESA  3UF7A  3UFEA  3UFFA  3UI4A  3UIDA  3UJCA  3UJIP  3UK0A  3ULTA  3UNVA  3UP3A  3UPLA  3UPVA  3UR8A  3URRA  3UUEA  3UV9A  3UW3A  3UXJA  3UZQB  3V0DA  3V1AA  3V30A  3V43A  3V46A  3V4GA  3V4KA  3V4NA  3V5CA  3V68A  3V75A  3V7NA  3V7QA  3V9OA  3VA4A  3VC5A  3VCAA  3VCXA  3VE9A  3VEJA  3VENA  3VG7A  3VGIA  3VGLA  3VI6A  3VIIA  3VJ9A  3VK5A  3VL1A  3VL9A  3VLAA  3VMKA  3VMNA  3VMVA  3VN0A  3VN3A  3VO2A  3VORA  3VQJA  3VRCA  3VRDA  3VRPA  3VSVA  3VTOA  3VUPA  3VURA  3VV1A  3VVIA  3VVVA  3VWCA  3VWNX  3VX0A  3VYKA  3VYPA  3VZXA  3W01A  3W06A  3W07A  3W0KA  3W0OA  3W0TA  3W19C  3W25A  3W56A  3W5SA  3W6WA  3W7TA  3W7YA  3WA2X  3WASA |
| --- | --- | --- | --- | --- | --- | --- | --- | --- | --- | --- | --- | --- | --- | --- | --- |

**Table S1.** (Continued)

| 3WCZA  3WDFA  3WDQA  3WEOA  3WG3A  3WGXA  3WH1A  3WH2A  3WIWA  3WJ1A  3WJPA  3WJTA  3WKGA  3WKQA  3WLIA  3WMQA  3WMTA  3WMVA  3WN7B  3WN8A  3WNDA  3WP4A  3WP9A  3WPCA  3WPUA  3WQBA  3WQCA  3WS7A  3WUCA  3WUPA  3WURA  3WUZA  3WV7A  3WVAA  3WVSA  3WWCA  3WWLA  3WWXA  3WX7A  3WY2A  3WYDA  3WYEA  3WZ1A  3WZ3A  3X0IA  3X0TA  3X2MA  3X34A  3X3YA  3ZBDA  3ZBOB  3ZDBA  3ZFPA  3ZHIA  3ZHNA  3ZHOA  3ZITA  3ZIYA  3ZMRA  3ZN4A  3ZN6A  3ZNVA  3ZOJA  3ZOQB  3ZPYA  3ZQXA  3ZR8X  3ZSUA  3ZTPA  3ZTVA  3ZUCA  3ZUZA  3ZVSA  3ZW5A  3ZXCA  3ZXFA  3ZXKA  3ZXYA  3ZY2A  3ZY7A  3ZYPA  3ZYQA  3ZZOA  3ZZPA  3ZZSA  3ZZYA  4A02A  4A14A  4A29A  4A2VA  4A37A  4A3PA  4A3ZA  4A41A  4A42A  4A4JA  4A4YA  4A6HA  4A6QA  4A6RA | 4A8TA  4A9VA  4AAAA  4AAZA  4AB4A  4AC1X  4ACJA  4AE7A  4AE8A  4AF8A  4AFFA  4AFMA  4AG1A  4AG7A  4AGIA  4AJ8A  4AK2A  4AK8A  4AL0A  4ALZA  4AMMA  4ANNA  4AO6A  4AO9A  4AOHA  4ARUA  4ASLA  4ASMB  4AT0A  4ATEA  4AU1A  4AUCA  4AUMA  4AVRA  4AVSA  4AW7A  4AWEA  4AWTA  4AX1B  4AXIA  4AXOA  4AXYA  4AY0A  4AYOA  4AZ6A  4B0HA  4B0ZA  4B15A  4B1MA  4B1YM  4B21A  4B4DA  4B4HA  4B4UA  4B5NA  4B5OA  4B6GA  4B6MA  4B89A  4B8VA  4B8XA  4B9GA  4B9IA  4B9PA  4BATA  4BB9A  4BBOA  4BEGA  4BEUA  4BF5A  4BFHA  4BGBA  4BGCA  4BGUA  4BH5A  4BI6A  4BJ0A  4BJIA  4BJZA  4BM1A  4BMHA  4BMNA  4BN4A  4BNDA  4BOQA  4BOUA  4BPFA  4BPSA  4BPYA  4BPZA  4BQNA  4BRCA  4BT7A  4BU0A  4BVXA  4BY8A  4BYZA  4BZ4A  4C08A  4C0XA | 4C1AA  4C24A  4C2VA  4C5CA  4C5KA  4C6AA  4C72A  4C81A  4CA1A  4CAYA  4CBPA  4CC2A  4CD5A  4CD8A  4CDJA  4CDPA  4CE8A  4CFIA  4CFQQ  4CGOA  4CGSA  4CHBA  4CHIA  4CI7A  4CI9A  4CICA  4CILA  4CJ0A  4CK4A  4CNDA  4CNGA  4CNNA  4COGA  4COQA  4CP6A  4CRGA  4CRQA  4CS4A  4CSRA  4CUAA  4CV7A  4CVRA  4CW4A  4CXPA  4CZ5A  4CZGA  4D0PA  4D0QA  4D6GA  4D74A  4D7JA  4D7UA  4D8BA  4DB5A  4DD5A  4DF0A  4DGFA  4DI9A  4DJAA  4DJCB  4DK2A  4DKNA  4DM5A  4DM7A  4DMIA  4DMVA  4DN2A  4DN7A  4DNDA  4DNXA  4DO4A  4DOIA  4DPBX  4DPZX  4DQ6A  4DQ9A  4DQAA  4DR8A  4DT4A  4DT5A  4DUIA  4DUQA  4DVCA  4DWDA  4DWOA  4DWRB  4DXKA  4DYEA  4DYQA  4DZHA  4DZIA  4E15A  4E19A  4E29A  4E2BA  4E2UA  4E2XA  4E3XA  4E40A  4E4RA | 4E4TA  4E4UA  4E69A  4E6UA  4E74A  4E9OX  4E9SA  4E9XA  4EA9A  4EADA  4EAEA  4EBGA  4EBJA  4ECFA  4EDHA  4EE6A  4EE9A  4EEKA  4EEWA  4EF0A  4EFIA  4EFPA  4EHUA  4EIEA  4EIHA  4EIRA  4EISA  4EIVA  4EKFA  4EMNA  4EO7A  4EP4A  4EQ9A  4EQAC  4EQBA  4EQPA  4EQSA  4ERCA  4ERRA  4ES1A  4ES8A  4ESMA  4ESRA  4ESUA  4ESWA  4ETMA  4ETNA  4EU9A  4EUNA  4EUOA  4EUZA  4EVQA  4EVUA  4EVZA  4EX6A  4EXKA  4EYSA  4EYZA  4EZFA  4EZIA  4F01A  4F06A  4F0BA  4F0WA  4F14A  4F1VA  4F2EA  4F2FA  4F2LA  4F3JA  4F66A  4F6EA  4F87A  4F8LA  4F8XA  4FAYA  4FAZA  4FB2A  4FBJA  4FCHA  4FFLA  4FGLA  4FH0A  4FK9A  4FKBA  4FN7A  4FNVA  4FOJA  4FS7A  4FZLA  4FZPA  4G0XA  4G1QB  4G3BA  4G3NA  4G3OA  4G41A  4G4GA  4G4KA  4G4PA | 4G4XA  4G54A  4G6CA  4G6TA  4G78A  4G7XA  4G9EA  4G9SA  4GA2A  4GB7A  4GBUA  4GC3A  4GCIA  4GCOA  4GE6A  4GEIA  4GEKA  4GGCA  4GGFA  4GHNA  4GIEA  4GJZA  4GMUA  4GNEA  4GNRA  4GOFA  4GOSA  4GQ4A  4GQMA  4GRZA  4GS3A  4GT8A  4GT9A  4GUCA  4GV1A  4GVFA  4GVOA  4GVQA  4GVXA  4GWBA  4GWGA  4GWIA  4GXWA  4GYMA  4GYXA  4H14A  4H15A  4H27A  4H2GA  4H3UA  4H4DA  4H4NA  4H53A  4H5IA  4H6CA  4H6QA  4H7PA  4H7UA  4H87A  4H89A  4H8EA  4HA4A  4HBQA  4HBZA  4HC5A  4HC9A  4HCJA  4HCSA  4HDDA  4HDEA  4HDRA  4HDTA  4HFQA  4HFSA  4HG2A  4HGUA  4HHRA  4HI7A  4HI8A  4HJIA  4HLSA  4HLYA  4HMSA  4HMWA  4HN1A  4HNLA  4HNOA  4HOJA  4HP8A  4HPNA  4HQ1A  4HQSA  4HROA  4HS1A  4HS2A  4HSTA  4HTFA  4HTGA  4HVKA  4HWMA | 4HWVA  4HY4A  4HZ2A  4HZ8A  4HZRA  4I0WA  4I1FA  4I1KA  4I3BA  4I3GA  4I4EA  4I62A  4I66A  4I6RA  4I6XA  4I6YA  4I71A  4I7WA  4I84A  4I8HA  4I8IA  4I93A  4IAUA  4IC4A  4ICIA  4ICVA  4ID0A  4ID9A  4IDCA  4IEDA  4IEJA  4IFAA  4IGIA  4IGJA  4IHEA  4IHMA  4IHZA  4IIKA  4IILA  4IIYA  4IJ5A  4IKCA  4IKDA  4IKEA  4IN0A  4IN9A  4INEA  4INKA  4INWA  4IO2A  4IPUA  4IQBA  4IQYA  4IRXA  4ITBA  4ITCA  4ITUA  4IUMA  4IUSA  4IX3A  4IX7A  4IYAA  4IYJA  4IZBA  4J0DA  4J0EA  4J1OA  4J20A  4J27A  4J3VA  4J42A  4J44A  4J4ZA  4J5RA  4J6OA  4J73A  4J7AA  4J7NA  4J7QA  4J8CA  4J8SA  4J9TA  4J9YB  4JB3A  4JB7A  4JBBA  4JBDA  4JCCA  4JDCA  4JDUA  4JE1A  4JEDA  4JEJA  4JEMA  4JERA  4JF1A  4JG2A  4JGIA  4JGLA  4JGUA | 4JHTA  4JIUA  4JJ7A  4JJAA  4JK8A  4JN7A  4JNUA  4JOKA  4JOSA  4JP6A  4JQFA  4JTMA  4JVUA  4JWXA  4JXRA  4JZ5A  4JZZA  4K0NA  4K12A  4K3FA  4K3LA  4K7BA  4K7ZA  4K82A  4K8GA  4K8YA  4K9QA  4KALA  4KAVA  4KBXA  4KDWA  4KDXA  4KEFA  4KEMA  4KG7A  4KGDA  4KH7A  4KH8A  4KL0A  4KLIA  4KLXA  4KM6A  4KMGA  4KMRA  4KN8A  4KNKA  4KQDA  4KQIA  4KQPA  4KRUA  4KS7A  4KT3A  4KU0A  4KU4A  4KUKA  4KV7A  4KXVA  4KZKA  4L05A  4L2HA  4L2IA  4L4EA  4L57A  4L58A  4L5EA  4L6DA  4L7XA  4L8AA  4L9DA  4L9OA  4L9PA  4LA2A  4LA9A  4LC3A  4LD1A  4LDCA  4LDVA  4LEBA  4LERA  4LF0A  4LGJA  4LGTD  4LGYA  4LHSA  4LIXA  4LIZA  4LJIA  4LJOA  4LKSA  4LKUA  4LLDA  4LLSA  4LLYA  4LMSA  4LMYA  4LOWA  4LPLA  4LPQA  4LR2A  4LRQA | 4LRTA  4LRUA  4LTTA  4LUAA  4LUKA  4LVUA  4LWLA  4LX2A  4LX3A  4LX4A  4LXQA  4LY1A  4LYPA  4LZXB  4M02A  4M0KA  4M0WA  4M1GA  4M1QA  4M1UA  4M1XA  4M2MA  4M51A  4M5EA  4M5RA  4M7TA  4M82A  4M8AA  4M91A  4M9KA  4M9VC  4MAGA  4MAIA  4MAKA  4MAMA  4MAQA  4MB1A  4MBYA  4MC3A  4MCKA  4MCOA  4ME2A  4MF5A  4MFIA  4MHPA  4MIJA  4MIYA  4MJDA  4MJEA  4MK3A  4MKXA  4MLLA  4MLVA  4MMGA  4MNCA  4MNKA  4MNOA  4MQ3A  4MQBA  4MTHA  4MTMA  4MTUA  4MUPA  4MUQA  4MUVA  4MUZA  4MVAA  4MX6A  4MXTA  4MYDA  4MYKA  4MYZA  4MZDA  4MZJA  4N02A  4N03A  4N0KA  4N13A  4N1IA  4N1VA  4N2KA  4N2PA  4N30A  4N3TA  4N4UA  4N5MA  4N67A  4N6CA  4N6KA  4N6XA  4N7FA  4N8CX  4N8GA  4NAZA  4NBRA  4NBTA  4NBUA  4NDOA  4NDSA  4NECA | 4NESA  4NETA  4NF1A  4NFNA  4NG0A  4NI6A  4NKPA  4NL9A  4NLMA  4NMUA  4NMWA  4NN2A  4NN3A  4NNOA  4NOAA  4NOBA  4NOGA  4NOHA  4NOVA  4NPDA  4NQ8A  4NSMA  4NSNA  4NSVA  4NTDA  4NTKA  4NUHA  4NUTA  4NX1A  4NXYA  4NYHA  4NYQA  4NZJA  4NZKA  4NZUL  4O06A  4O0AA  4O0CA  4O0KA  4O4VA  4O59O  4O5FA  4O6UA  4O7HA  4OA3A  4OANA  4OB0B  4OCVA  4OD6A  4ODKA  4OE9A  4OELA  4OFAA  4OGDB  4OGGA  4OH7A  4OHJA  4OHNA  4OI3A  4OIYA  4OJXA  4OKIA  4OLTA  4OM8A  4OMBA  4ONMA  4ONRA  4OPCA  4OQ9A  4OQPA  4OQVA  4OTKA  4OU0A  4OUJA  4OUSA  4OX6A  4OXXA  4OY3A  4OY7A  4OZJA  4P0LA  4P0TA  4P0ZA  4P32A  4P3AA  4P3HA  4P3VA  4P40A  4P47A  4P5EA  4P5NA  4P5PA  4P7OA  4P7XA  4P82A  4P8BA  4P9IA  4PAKA  4PBHA  4PC9A | 4PCAA  4PDNA  4PDYA  4PE0X  4PE3A  4PF3A  4PF4A  4PF8A  4PFYA  4PH2A  4PH8A  4PHJA  4PHRA  4PI8A  4PICA  4PIOA  4PITA  4PJ2A  4PKLA  4PLZA  4PMOA  4PMXA  4PNEA  4PNOA  4POWA  4PP4A  4PQ9A  4PQDA  4PQHA  4PQQA  4PS6A  4PSCA  4PSFA  4PSRA  4PSSA  4PUXA  4PVAA  4PVKA  4PW0A  4PWOA  4PWWA  4PXEA  4PXYA  4PYRA  4PZ0A  4PZ3A  4PZJA  4PZKA  4Q27A  4Q29A  4Q2LA  4Q2QA  4Q2SA  4Q34A  4Q3KA  4Q4GX  4Q4W1  4Q68A  4Q6JA  4Q6TA  4Q7EA  4Q7OA  4Q7QA  4Q7ZA  4Q98A  4Q9BA  4QA8A  4QA9A  4QASA  4QB3A  4QB6A  4QBOA  4QC6A  4QDJA  4QEKA  4QF3A  4QGOA  4QHEA  4QHPA  4QHQA  4QI3A  4QI8A  4QITA  4QKDA  4QLPA  4QM6A  4QNSA  4QOSA  4QP5A  4QPNA  4QPTA  4QPWA  4QQHA  4QQSA  4QRNA  4QT3A  4QTCA  4QUCA  4QUSA  4QWOA | 4QXBB  4QXLA  4QY7A  4QYOA  4QYTA  4QYWA  4R16A  4R1JA  4R1SA  4R1VA  4R2XC  4R38A  4R3FA  4R3NA  4R52A  4R5RA  4R6HA  4R6RA  4R6YA  4R75A  4R78A  4R81A  4R8HA  4R8XA  4R9FA  4R9PA  4RAXA  4RAYA  4RBXA  4RCJA  4RD4A  4RD7A  4RDBA  4REIA  4REKA  4REOA  4REXA  4RFUA  4RGDA  4RGYA  4RI5A  4RI6A  4RJ2A  4RJWA  4RJZA  4RK4A  4RKFA  4RL3A  4RLCA  4RLEA  4RLZA  4RP3A  4RPMA  4RPTA  4RQAA  4RQRA  4RRIA  4RS2A  4RT5A  4RTHA  4RU1A  4RU3A  4RU5A  4RUQA  4RUWA  4RV5A  4RVQA  4RWCA  4RWUA  4RXLA  4RXTA  4RXUA  4RXVA  4RY1A  4RY9A  4RYAA  4RYOA  4RZ9A  4S12A  4S1HA  4S1PA  4S28A  4S2XA  4S35A  4S36A  4S39A  4S3JA  4TKBA  4TKCA  4TM7A  4TMXA  4TOZA  4TPNA  4TPVA  4TQRA  4TQXA  4TR1A  4TR6A  4TROA  4TSDB | 4TTNA  4TTWA  4TVVA  4TXRA  4TXWA  4TYZA  4TZ1A  4TZHA  4U0OB  4U36A  4U3YA  4U5HA  4U5RA  4U7AA  4U89A  4U8FA  4U98A  4U9HL  4U9OA  4U9UA  4UA6A  4UA8A  4UABA  4UASA  4UAVA  4UDGA  4UDQA  4UDXX  4UE0A  4UE8A  4UFQA  4UHCA  4UHOA  4UHQA  4UHTA  4UIQA  4UJ7A  4ULVA  4UMIA  4UN2B  4UNUA  4UOBA  4UP0A  4UP3A  4UPIA  4UQWA  4UQXA  4UQZB  4URFA  4USAA  4USIA  4UTOA  4UTUA  4UU3A  4UU5A  4UULA  4UWWA  4UYBA  4UYPA  4UYRA  4UZGA  4V0KA  4V0WB  4V12A  4V15A  4V1GA  4V1JA  4V1KA  4V1SA  4V29A  4V33A  4V3IA  4V3LC  4W5ZA  4W64A  4W6YA  4W78A  4W79A  4W7LA  4W7WA  4W88B  4W8BA  4W8HA  4W8PA  4W8QA  4W9ZA  4WBJA  4WBTA  4WBYA  4WCGA  4WCKA  4WCXA  4WDCA  4WE2A  4WECA  4WEEA  4WEPA  4WESA  4WF5A  4WFOA | 4WFVA  4WH9A  4WHSA  4WILA  4WIQA  4WJIA  4WJOB  4WJQA  4WJTA  4WK7A  4WKAA  4WLHA  4WN5A  4WNDB  4WNOA  4WOHA  4WP9A  4WPGA  4WPKA  4WPYA  4WQDA  4WQKA  4WRIA  4WSFA  4WTPA  4WTXA  4WU0A  4WUIA  4WUTA  4WUVA  4WWFA  4WWHA  4WXTA  4WY4A  4WY9A  4WYDA  4WZXA  4X00A  4X1ZA  4X2RA  4X33A  4X54A  4X5PA  4X7GA  4X7YB  4X84A  4X8EA  4X9CA  4X9JA  4X9RA  4X9TA  4X9XA  4X9ZA  4XA7A  4XBAA  4XCBA  4XCVA  4XD1A  4XDUA  4XDXA  4XDYA  4XDZA  4XEDA  4XEMA  4XEPA  4XEZA  4XFEA  4XFJA  4XFKA  4XFMA  4XFWA  4XHVA  4XHYA  4XIJA  4XINA  4XJ5A  4XJWA  4XKBA  4XLZA  4XMRA  4XOSA  4XOTA  4XPXA  4XPZA  4XQ7A  4XQCA  4XRMA  4XSLA  4XTBA  4XTLA  4XTVA  4XUWA  4XXFA  4XXLA  4XXXA  4XYBA  4XZFA  4XZPA  4Y1BA  4Y1WA | 4Y2FA  4Y2MA  4Y31A  4Y6WA  4Y7LA  4Y88A  4Y8FA  4Y96A  4Y9IA  4Y9MA  4Y9VA  4Y9WA  4YAAA  4YAGA  4YAPA  4YBMA  4YCBA  4YDRA  4YE7A  4YECA  4YEPA  4YFMA  4YFUA  4YG0A  4YGBB  4YI8A  4YJRA  4YKIA  4YL8A  4YLAA  4YLQT  4YMEA  4YMXA  4YMYA  4YNHA  4YNUA  4YNXA  4YORA  4YPOA  4YQDA  4YQYA  4YSIA  4YSLA  4YTBA  4YTDA  4YTKA  4YTWA  4YUCA  4YUDA  4YVOA  4YWAA  4YWKA  4YX1A  4YYCA  4YYUA  4YZ0A  4YZGA  4YZNA  4YZRA  4YZZA  4Z04A  4Z0GA  4Z0OA  4Z0TA  4Z0WA  4Z0YA  4Z1RA  4Z2OA  4Z39A  4Z3GA  4Z47A  4Z4DA  4Z4JA  4Z55A  4Z65A  4Z67A  4Z6MA  4Z79A  4Z7EA  4Z7XA  4Z80A  4ZA9A  4ZAVA  4ZBDA  4ZBGA  4ZBHA  4ZBLA  4ZBOA  4ZC3A  4ZCEA  4ZD6A  4ZDMA  4ZEYA  4ZFVA  4ZGFA  4ZGWA  4ZH5A  4ZHBA  4ZHWA  4ZILA | 4ZJHA  4ZL8A  4ZLDA  4ZLFA  4ZMKA  4ZMYA  4ZO2A  4ZOTA  4ZOXA  4ZOYA  4ZPCA  4ZQXA  4ZR8A  4ZRXA  4ZS9A  4ZURA  4ZV0A  4ZV5A  4ZVCA  4ZVFA  4ZW9A  4ZWVA  4ZX2A  4ZXOA  4ZY9A  4ZZ1A  4ZZNA  5A0DA  5A0LA  5A0NA  5A0YA  5A10A  5A12A  5A1IA  5A35A  5A3AA  5A57A  5A61A  5A62A  5A67A  5A6MA  5A71A  5A7GA  5A7VA  5A8CA  5A8JA  5A95A  5A99A  5A9TA  5AB8A  5ABWA  5ACHA  5ACSA  5AD1A  5AE0A  5AEZA  5AFWA  5AFYH  5AGDA  5AGIA  5AGRA  5AH1A  5AHKA  5AIGA  5AIHA  5AILA  5AIMA  5AJGA  5AJOA  5AKRA  5AL6A  5AL9A  5AMVA  5AN5B  5ANPA  5AO9A  5AOGA  5AOVA  5APGA  5APHA  5AQ0A  5AR6A  5AULA  5AVDA  5AWOA  5AX0A  5AZBA  5AZWA  5AZXA  5B08A  5B1AA  5B1RA  5B1SA  5B4BA  5B4TA  5B4ZA  5B5IA  5B5LA  5B5ZA  5B6CA | 5B78B  5B7GA  5B7HA  5B7YA  5B89A  5B8DA  5BJXA  5BK6A  5BMNA  5BMTA  5BOBA  5BOEA  5BOVA  5BOWA  5BP3A  5BP9A  5BPKA  5BR4A  5BS1A  5BSRA  5BT9A  5BTWA  5BTYA  5BV8A  5BWIA  5BXOA  5BXRA  5BY5A  5BY8A  5BYKA  5C04A  5C0PA  5C12A  5C17A  5C2UA  5C30A  5C33A  5C3FB  5C40A  5C4MA  5C5GA  5C5TA  5C5ZA  5C68A  5C6SA  5C79A  5C7HA  5C7QA  5C86A  5C8ZA  5C98A  5C9OA  5CADA  5CB7A  5CD2A  5CDKA  5CDVA  5CECA  5CEGA  5CFJA  5CG5A  5CGOA  5CGQA  5CIVA  5CIYA  5CKLA  5CL8A  5CM7A  5CMLA  5COFA  5COWA  5COYA  5COZA  5CPHA  5CQ2A  5CR4A  5CRWA  5CTAA  5CTDC  5CTMA  5CTVA  5CUOA  5CVDA  5CVWA  5CWBA  5CWGA  5CWHA  5CWLA  5CXXA  5CYVA  5CZWA  5D1MB  5D2KA  5D4NA  5D4VA  5D5YB  5D66A  5D6EA  5D78A  5D7UA |
| --- | --- | --- | --- | --- | --- | --- | --- | --- | --- | --- | --- | --- | --- | --- | --- |

**Table S1.** (Continued)

| 5D7WA  5D84A  5D8VA  5D94A  5D9OA  5DAEA  5DAGA  5DBLA  5DCLA  5DCUA  5DE3A  5DFYA  5DGJA  5DHDA  5DICA  5DJHA  5DKAA  5DKXA  5DLDA  5DLEA  5DLOA  5DLTA  5DLYA  5DM2A  5DMAA  5DMDA  5DNLA  5DP2A  5DTKA  5DU9A  5DUFA  5DUSA  5DUTA  5DVIA  5DVXA  5DWAB  5DXLA  5DXXA  5DZDA  5DZEA  5DZOA  5DZSA  5E1NA  5E1WA  5E1YA  5E37A  5E4BA  5E4GA  5E56A  5E5YA  5E68A  5E75A  5E7HA  5E8SA  5E95B  5E9PA  5EC6A  5ECKA  5EDFA  5EEQA  5EHAA  5EHIA  5EJ8A  5EL3A  5EL9A  5ELBA  5EM0A  5EMBA  5EMIA  5EMXA  5ENFA  5EP2A  5EP6A  5EPBA  5EPFA  5EPWA  5EQ0A  5EQ7A  5EQVA  5ER6A  5ETRB  5EU0A  5EW0A  5EWOA  5EWUA  5EWYA  5EX2A  5EXHC  5EY0B  5EYFA  5EYNA  5EZUA  5F07A  5F23A  5F2KA  5F3MA  5F47A  5F4CA  5F5NA  5F68A | 5F6EA  5F6RA  5F7FA  5F7VA  5F82A  5FA8A  5FAAA  5FAFA  5FAGA  5FAVA  5FBFA  5FC1A  5FC9A  5FD9A  5FEBA  5FEWA  5FFDA  5FFFA  5FFXA  5FG6A  5FH7A  5FI3A  5FISA  5FJDA  5FJLA  5FLWA  5FLYA  5FMUA  5FOCA  5FPZA  5FQAA  5FQEA  5FRDA  5FS8A  5FSVA  5FTBA  5FTZA  5FU5A  5FUIA  5FUKA  5FV5A  5FVNA  5FX6A  5FYDA  5FYPA  5G08A  5G0GA  5G1AA  5G28A  5G2UA  5G2VA  5G38A  5G3YA  5G4IA  5G51A  5G5CA  5GGBA  5GGNA  5GI7A  5GIZA  5GJIA  5GJUA  5GKMA  5GLJA  5GM9A  5GMDA  5GNFA  5GNGA  5GQIA  5GRQA  5GS7A  5GS8A  5GSMA  5GT5A  5GTQA  5GTUA  5GUAA  5GV0A  5GV8A  5GVRA  5GWNA  5GXXA  5GY6A  5GY7A  5GYCA  5GZ3A  5GZCA  5H0MA  5H0QA  5H1NA  5H28A  5H2DA  5H3GA  5H3JA  5H3VA  5H6TA  5H6XA  5H7EA  5H7KA  5H9IA | 5H9NA  5HB6A  5HB7A  5HBPA  5HBSA  5HC0A  5HDIA  5HDKA  5HDMA  5HEEA  5HFKA  5HGJA  5HGWA  5HGZA  5HHAA  5HHED  5HHJA  5HJ1A  5HJ9A  5HJFA  5HK3A  5HKOA  5HL3A  5HMLA  5HNVA  5HOEA  5HPJA  5HQHA  5HQJA  5HRAA  5HSFA  5HSGA  5HT2A  5HTLA  5HTXA  5HUBA  5HW3A  5HWAA  5HWKA  5HWNA  5HWOA  5HZ5A  5HZDA  5I0YA  5I1UA  5I29A  5I2HA  5I32A  5I34A  5I39A  5I45A  5I55A  5I5BA  5I5MA  5I5NA  5I7IA  5I86A  5I8FA  5I90A  5I95A  5IAIA  5IB9A  5IBQA  5ICEA  5ICUA  5IDBB  5IDHA  5IDQA  5IDVA  5IFZA  5IG6A  5IGIA  5IHFA  5IHSA  5IHVA  5IHWA  5II6A  5II8A  5IJMA  5IK4A  5IMAA  5IN1A  5INBA  5IO9A  5IPYA  5IQNA  5IR4A  5ISVA  5IT3A  5IT6A  5ITMA  5ITQA  5ITWA  5IU0A  5IUCA  5IVKA  5IWHA  5IWUA  5IX8A  5IXBA | 5IXGB  5IXHA  5IY2A  5IZ3A  5IZAA  5J0KA  5J1JA  5J1NA  5J1SA  5J3TA  5J41A  5J4FA  5J4LA  5J4OA  5J4UA  5J6YA  5J80A  5J90A  5JAJA  5JAWA  5JAZA  5JB9E  5JBNA  5JBTY  5JBXA  5JC8A  5JCAL  5JDAA  5JDDA  5JDKA  5JE2A  5JELA  5JFWA  5JGKA  5JGYA  5JH8A  5JHXA  5JI7A  5JICA  5JIGA  5JIXA  5JJ2A  5JK4A  5JKJA  5JLAA  5JLBA  5JNTA  5JO8A  5JODA  5JOVA  5JOWA  5JP6A  5JPHA  5JQFA  5JQNA  5JRTA  5JRYA  5JS4A  5JSCA  5JSKA  5JUGA  5JUHA  5JVIE  5JVVB  5JXMA  5K08A  5K26A  5K2IA  5K2LA  5K2XA  5K34A  5K3XA  5K4BA  5K6DA  5K79A  5K7AA  5K86A  5K87A  5K8JA  5K8SA  5K91A  5K9BA  5KARA  5KB6A  5KDIA  5KDSA  5KF9A  5KFZA  5KHTA  5KI9A  5KJZA  5KKOA  5KLAA  5KLEA  5KNHI  5KO4A  5KO5A  5KO9A  5KP7A  5KPGA | 5KTNA  5KVBA  5KVCA  5KVGE  5KVRA  5KVSA  5KWMA  5KXHA  5KY4B  5KYCB  5KZZA  5L0NA  5L0VA  5L20A  5L2LA  5L37A  5L4LA  5L6UA  5L74A  5L77A  5L87A  5L9AA  5L9ZA  5LALA  5LAUA  5LB7A  5LBDA  5LDGA  5LEOA  5LF9A  5LFZA  5LHMA  5LHWA  5LHXA  5LI7A  5LJLA  5LJMA  5LJPA  5LJXA  5LKBA  5LNDA  5LNNA  5LOMA  5LP0A  5LP9A  5LPAA  5LQ5A  5LQ6A  5LS4A  5LS7A  5LSVA  5LT5A  5LTLA  5LU5A  5LUNA  5LUSA  5LVOA  5LW3A  5LWXA  5LX6A  5LXEA  5LXXA  5LXZB  5LY3A  5LY8A  5LYPA  5LZKA  5LZNA  5M0NA  5M0WA  5M0YA  5M10A  5M17A  5M1MA  5M1PA  5M29A  5M2OB  5M2PA  5M33A  5M3QA  5M4BA  5M4VA  5M5ZA  5M72A  5M7YA  5M97B  5MAOA  5MAWD  5MBXA  5MC1A  5MC7A  5MDUA  5MFAA  5MFOA  5MGWA  5MH6A  5MJRA  5MK9A  5ML3B  5MLNA | 5MOZA  5MPRA  5MPWA  5MQ5A  5MR1A  5MSAA  5MSOA  5MSZA  5MTEA  5MU9A  5MUAA  5MUJA  5MULA  5MWAA  5MX9A  5MXCA  5MXPA  5MY5A  5MY7A  5MYCP  5MZWA  5N0OA  5N13A  5N17A  5N1PA  5N3JA  5N41A  5N48B  5N4BA  5N4IA  5N4KA  5N5DA  5N5UA  5N6FA  5N7QA  5N81A  5N86A  5N8AX  5NAIA  5NAKA  5NB4A  5NCBA  5NCGA  5NCJA  5NCWA  5NE2A  5NFMA  5NFQA  5NG7A  5NG9A  5NGGA  5NGNA  5NHUI  5NI9A  5NIOA  5NJ2A  5NJ9A  5NJIA  5NJOA  5NLDA  5NMNA  5NMXA  5NMZA  5NNAA  5NOAA  5NQOA  5NR4A  5NRHA  5NRMA  5NS6A  5NSAA  5NT7A  5NTBA  5NULA  5NUVA  5NVGA  5NW3A  5NWGA  5NWPA  5NX7A  5NYKA  5NZ4A  5NZGA  5NZOB  5O0SA  5O0UA  5O15A  5O1LA  5O1XA  5O29A  5O2DA  5O2XA  5O37A  5O45A  5O58A  5O5SA  5O63A  5O6HA  5O6TA  5O75A | 5O95A  5O99B  5O9MA  5O9QA  5OAKA  5OAVA  5OAZA  5OBPA  5OBTA  5OBYA  5OD4A  5ODJA  5ODKA  5ODUA  5OE3A  5OF1A  5OFKA  5OGZA  5OHQA  5OJ5A  5OJ7A  5OJCA  5OJIA  5OJLA  5OK4A  5OK6A  5OKAA  5OL4A  5OL9A  5OLLA  5OLRA  5OLTA  5OMTA  5ON8A  5ONKA  5ONNA  5OOXA  5OPFA  5OPZA  5OQ3A  5OTNA  5OUOA  5OVKA  5OVOA  5OVVA  5OXZA  5OYCA  5P9JA  5P9VA  5PAXA  5PMXA  5Q22A  5QHHA  5QI0A  5QINA  5QOQA  5QR1A  5SUIA  5SV2A  5SV5A  5SVYA  5SWCA  5SXOA  5SY4A  5SZCA  5SZDA  5T1IA  5T39A  5T3BA  5T46B  5T7AA  5T7DA  5T8CA  5T9CE  5TA0A  5TABA  5TC6A  5TCBA  5TDAA  5TDRA  5TFQA  5TG0A  5THKA  5TIFA  5TJZA  5TK2A  5TKWA  5TKZA  5TLEA  5TNVA  5TNWA  5TOQA  5TPIA  5TQIA  5TQJA  5TRQA  5TSQA  5TT5A  5TUXA  5TV2A | 5TVOA  5TVYA  5TW4A  5TW9A  5TZ5A  5TZMA  5TZPA  5U00A  5U0IA  5U1HA  5U23C  5U2OA  5U3AA  5U3QA  5U4HA  5U4NA  5U4QA  5U4SA  5U5OA  5U5TC  5U69A  5U7AA  5U81A  5U8UA  5UAMA  5UBAA  5UCSA  5UDIA  5UE1A  5UEBA  5UEJA  5UF2A  5UFHA  5UFNA  5UFYA  5UG9A  5UGGA  5UGRA  5UJCA  5UJKA  5UL6A  5ULBA  5UM2A  5UMFA  5UMHA  5UMPA  5UMRA  5UMSA  5UOUA  5UQ6A  5UQSA  5UQZA  5UR4A  5UT3A  5UUIA  5UUKA  5UUOB  5UWAA  5UWZA  5UX1A  5UXMA  5UXSA  5UYJA  5UZGB  5UZMA  5UZXA  5V01A  5V0MA  5V0ZA  5V1VA  5V1YA  5V2OA  5V37A  5V3NA  5V44A  5V5HA  5V6FA  5V6JA  5V89A  5V8SA  5VBDA  5VCMA  5VCZA  5VEIA  5VEOA  5VFAA  5VFBA  5VG0A  5VG3A  5VGBA  5VGLA  5VHGA  5VI6A  5VIVA  5VJTA  5VN4A  5VNYA  5VOGA  5VPQA  5VPSA | 5VPUA  5VRKA  5VSCA  5VTJA  5VUGA  5VX1A  5VX5A  5VXVA  5VYQA  5VZBA  5W0GA  5W0HA  5W2FA  5W2IA  5W3RA  5W4AA  5W7WT  5W83A  5W89A  5W8JA  5W8MA  5W8OA  5W8QA  5W98A  5WA2A  5WD9A  5WECA  5WFBA  5WFYA  5WGIA  5WH8A  5WJPA  5WK0A  5WKRA  5WL1A  5WLJA  5WM2A  5WMKA  5WN9A  5WP4A  5WQJA  5WRIA  5WSFA  5WSLA  5WUCA  5WUTA  5WWDA  5WXHA  5X2EA  5X40A  5X4BA  5X4RA  5X57A  5X5HA  5X5JA  5X5MA  5X5VA  5X7LA  5X89A  5X9IA  5X9LA  5XA5A  5XAVA  5XB0A  5XBCA  5XBIA  5XBUA  5XC5A  5XCOB  5XCTA  5XDCA  5XDHA  5XDTA  5XECA  5XEVA  5XJ5A  5XK6A  5XKAA  5XKRA  5XKXA  5XLUA  5XM5A  5XN3A  5XN9A  5XNEB  5XTUA  5XVEA  5XVJA  5XVTA  5XW2A  5XWXA  5XXLA  5XZ4A  5XZ7A  5Y00A  5Y0MA  5Y1FA  5Y2SA  5Y33A  5Y37A | 5Y46A  5Y4MA  5Y4TA  5Y4ZA  5Y5QA  5Y6UA  5Y6YB  5Y90A  5Y9XA  5Y9ZA  5YA6A  5YALA  5YAYA  5YBYA  5YC6U  5YCAA  5YCEA  5YDDA  5YDEA  5YDNA  5YEDA  5YGBA  5YH4A  5YHRA  5YIUA  5YJ6A  5YKJA  5YKRA  5YKUA  5YKZA  5YL7A  5YLGA  5YMXA  5YNXA  5YOBA  5YOFA  5YQAA  5YQJA  5YQWA  5YRHA  5YRVA  5YSEA  5YSIA  5YSQA  5YT6F  5YTXA  5YUFA  5YUGA  5YUQA  5YVKA  5YVNA  5YVXA  5YWRB  5YXGA  5YXMA  5YZPA  5Z0DA  5Z0UA  5Z1BA  5Z37A  5Z3EA  5Z42A  5Z48A  5Z4GA  5Z51A  5Z6BA  5Z6DA  5Z99A  5Z9YA  5ZA3A  5ZB0A  5ZBFA  5ZBYA  5ZBZA  5ZCEA  5ZCYA  5ZDAA  5ZDMA  5ZE8A  5ZGMA  5ZGXA  5ZHOA  5ZHZA  5ZIMA  5ZIQA  5ZKEB  5ZM0A  5ZMUA  5ZO3A  5ZOHA  5ZQAA  5ZRCA  5ZRQA  5ZRXA  5ZRYA  5ZT3A  5ZTDA  5ZU6A  5ZW7A  5ZWUA | 5ZX8A  5ZX9A  5ZZAP  5ZZRA  6A02A  6A0JA  6A1AA  6A1IA  6A27A  6A2QA  6A56A  6A66A  6A71A  6A7TB  6A80A  6A8KA  6A8RB  6A9SA  6AC0A  6AC5A  6AE9A  6AIBA  6AJPA  6AKKA  6AM3X  6AMGA  6ANZA  6AO9A  6APEA  6AQSA  6AR0A  6ARHA  6AROA  6AT0A  6AT4A  6ATRA  6ATWA  6AVXA  6AYMA  6AZMF  6B1KA  6B1ZA  6B26A  6B29A  6B2VA  6B4PA  6B5KA  6B6UA  6B7PA  6B8FA  6B9HA  6B9XA  6BA9A  6BCBA  6BCDA  6BD0A  6BDNA  6BEVA  6BG8A  6BGDA  6BGNA  6BGYA  6BHDA  6BHKA  6BIOA  6BJAA  6BJBA  6BK0A  6BLKC  6BLMA  6BM5A  6BNZA  6BO0A  6BQAA  6BSCA  6BSUA  6BT1A  6BW9A  6BWLA  6BXDA  6BXGA  6BXRA  6C10A  6C1XA  6C1ZA  6C29A  6C2CA  6C2ZA  6C30A  6C3CA  6C3MA  6C4QA  6C52A  6C5BA  6C74A  6C9EA  6C9XA  6CAFA  6CAXA  6CB4A | 6CB7A  6CBNA  6CBRA  6CBUA  6CD7A  6CD9A  6CHXA  6CJ7A  6CKAA  6CKMA  6CNWA  6COFA  6COJA  6CPBA  6CQPA  6CR0A  6CTZA  6CUMA  6CW0A  6CWMA  6CZ4A  6CZXA  6D0AA  6D0HA  6D2KA  6D4KA  6D4RA  6D9NA  6D9YA  6DCDA  6DCEA  6DCJA  6DCMA  6DDMC  6DFPA  6DG4A  6DGAA  6DGGA  6DGMA  6DHTA  6DKQA  6DNMA  6DNOB  6DOPA  6DQPA  6DRRA  6DS9A  6DSPA  6DT3A  6DTSA  6DTVA  6DUBA  6DVRA  6DYFA  6E0KA  6E0OA  6E1XA  6E1ZA  6E28C  6E3AA  6E3IB  6E4LA  6E55A  6E5FA  6E5XA  6E5YA  6E60A  6E68A  6E6NA  6E6OA  6E6QA  6E6UB  6E7EA  6E85A  6E94A  6EA2A  6ECTA  6EDVA  6EF6A  6EFNA  6EGED  6EH4D  6EHBA  6EHIA  6EI0Q  6EIMA  6EIOA  6EKGY  6EKLB  6EKZA  6ELCA  6ELMA  6ELVA  6ELWA  6ENIA  6ENPA  6ENSA  6EOZA  6EQEA  6EQMA | 6EQSA  6ER1A  6ER4A  6ER6A  6ERKA  6ES9A  6ESMA  6ET0A  6ET6A  6ETLA  6EU8A  6EUWA  6EVGA  6EVNA  6EVUA  6EWHA  6EWLA  6EWMA  6EXMA  6EXXA  6EXZA  6EY1A  6EYGA  6EZIA  6F0PA  6F0WS  6F43A  6F4JA  6F5CA  6F5ZA  6F6MA  6F6OA  6F70A  6F8AA  6F8BA  6F8NA  6F8PA  6F9MA  6F9OA  6F9QA  6FBCA  6FBQA  6FC0A  6FC1A  6FCHA  6FD3A  6FDGA  6FDKA  6FEAA  6FEXA  6FF1A  6FF2A  6FFAA  6FG8A  6FGCA  6FGGA  6FI2A  6FIEB  6FIHA  6FIYA  6FJ7A  6FJNA  6FJVA  6FKWA  6FL1A  6FLKA  6FM5A  6FM7A  6FMBA  6FMCA  6FMEB  6FN8A  6FNGA  6FNUA  6FOHA  6FOPA  6FOQA  6FPOL  6FPQA  6FQ1A  6FREA  6FRWA  6FSGA  6FSKA  6FSNA  6FT2A  6FTFB  6FTHA  6FTOA  6FU9A  6FUCA  6FVIA  6FW0A  6FXAA  6FXDA  6FXWA  6FYJA  6FYRA  6FZ6A  6G00A | 6G1CV  6G1IA  6G1PA  6G28A  6G44A  6G47A  6G49A  6G4JA  6G5PA  6G62A  6G65A  6G6KA  6G75A  6G7NA  6G85A  6G8UA  6G8YA  6G96B  6GAJA  6GAML  6GBCA  6GBIA  6GCFA  6GCVA  6GD3A  6GDJA  6GDXA  6GEHA  6GEUA  6GG1A  6GG7B  6GGPA  6GHTA  6GI2A  6GI4B  6GJFA  6GK5A  6GKXA  6GM5A  6GMCA  6GMFA  6GMPA  6GN5A  6GNAA  6GP3A  6GPKA  6GPUA  6GPZB  6GQDA  6GQZA  6GREA  6GRLA  6GS0A  6GSCB  6GSZA  6GUGA  6GV3A  6GVDA  6GVKB  6GX2A  6GY5A  6GZ0A  6GZ8A  6GZUA  6GZWA  6H0CA  6H0HA  6H0MA  6H1HA  6H1QA  6H20A  6H24A  6H2RC  6H2UA  6H40A  6H4EA  6H4LA  6H5VA  6H5WA  6H8OA  6H96A  6H99A  6H9UB  6HA4A  6HAZA  6HBBA  6HCNB  6HCWA  6HDTA  6HFCA  6HFMA  6HFQA  6HGMA  6HHEA  6HHMA  6HIHA  6HIPA  6HIUB  6HL1A  6HLYA | 6HMQA  6HPFA  6HPHA  6HS0A  6HSDA  6HSHA  6HSJA  6HTNF  6HTOA  6HXMA  6HXOA  6HXPA  6HY3A  6HYFA  6HYOA  6HYYA  6HZGA  6I18A  6I20D  6I3QA  6I4EA  6I4FA  6I4HA  6I5RA  6I6MA  6I6VB  6I8YA  6I9AA  6IBDA  6IBEA  6ICGA  6IDNA  6IF3A  6IH0A  6IHRA  6IIPA  6IJEB  6IQ9A  6IQCA  6IQXA  6ITAA  6ITGA  6J0EB  6J3PA  6J4KB  6J4PA  6J93A  6JAVA  6JCCA  6JD9A  6JEBA  6JEDA  6JGJA  6JI6A  6JKZA  6JLEA  6JNJA  6JPTA  6JVVA  6K4XA  6K5GA  6M7KA  6M80C  6M8NA  6M9MA  6M9ZA  6MAAA  6MAZC  6MB8A  6MBBB  6MBFA  6MDHA  6MDWA  6MFUA  6MGCA  6MIHA  6MJ7A  6MJKA  6MOGA  6MR1A  6MROA  6MRRA  6MSNA  6MSQA  6MU0A  6MU9A  6MVUA  6MWSA  6MX3A  6MYDA  6MYEA  6MYIA  6MYWA  6N0KA  6N0XC  6N19A  6N1BA  6N1MA  6N36A  6N3DA | 6N4LA  6N7AA  6N87A  6N8YA  6N9IA  6N9MA  6NAUA  6NAXA  6NDRA  6NE2A  6NFRA  6NHXA  6NIBA  6NIOA  6NJKA  6NK0A  6NKJA  6NNRA  6NPPA  6NQ6A  6NSVA  6NUPA  6NX0A  6NX5A  6NYOA  6NYTA  6NZSA  6O19A  6O2VA  6O3PA  6O4MA  6OD3A  6OHKA  6OJAF  6OJMA  6OLXA  6ON6A  6ONCD  6OS6A  6OSXA  6OVIA  6OXJA  6OZ7A  6P29B  6P2LA  6P2NA  6PFXA  6PNVA  6Q4GA  6Q4RA  6Q5OA  6Q6TA  6Q7IB  6Q7RA  6Q8EA  6Q8MA  6Q9LA  6QE0A  6QHGA  6QJLA  6QKUA  6QNVA  6QPRA  6QSPA  6QTSA  6QU7A  6QVFA  6R1DB  6R1HA  6R4ZA  6R62A  6R8RA  6REKA  6RHFA  6RI6A  6RIMA  6RK0A  6RLXC  6RXNA  6S1HA  7A3HA  7FD1A  7ODCA  8ABPA |
| --- | --- | --- | --- | --- | --- | --- | --- | --- | --- | --- | --- | --- | --- | --- | --- |

**Table S2.** The order of principal interactions extracted by different mean force potential functions.

| Potential function | DBNI | DFIRE | DOPE |
| --- | --- | --- | --- |
| 1 | LEU-LEU | LEU-LEU | LEU-LEU |
| 2 | CYS-CYS | LEU-ILE | LEU-VAL |
| 3 | PHE-LEU | LEU-VAL | LEU-ILE |
| 4 | VAL-VAL | VAL-VAL | PHE-LEU |
| 5 | LEU-ILE | ILE-ILE | ILE-ILE |
| 6 | PHE-PHE | PHE-LEU | VAL-VAL |
| 7 | ILE-ILE | PHE-PHE | VAL-ILE |
| 8 | LEU-VAL | ILE-VAL | ALA-LEU |
| 9 | ILE-VAL | LEU-ALA | PHE-PHE |
| 10 | LEU-TYR | ALA-ALA | ILE-PHE |
| 11 | PHE-ILE | PHE-ILE | TYR-LEU |
| 12 | PHE-VAL | PHE-VAL | PHE-VAL |
| 13 | TYR-TYR | LEU-TYR | VAL-ALA |
| 14 | LEU-TRP | VAL-ALA | CYS-CYS |
| 15 | ILE-TYR | ILE-ALA | ILE-ALA |
| 16 | LEU-ALA | TYR-TYR | TYR-TYR |
| 17 | PHE-TYR | PHE-TYR | PHE-TYR |
| 18 | VAL-TYR | CYS-CYS | ILE-TYR |
| 19 | VAL-ALA | VAL-TYR | VAL-TYR |
| 20 | TRP-PHE | ILE-TYR | LEU-THR |
| 21 | MET-LEU | PHE-ALA | PHE-ALA |
| 22 |  |  | TRP-LEU |


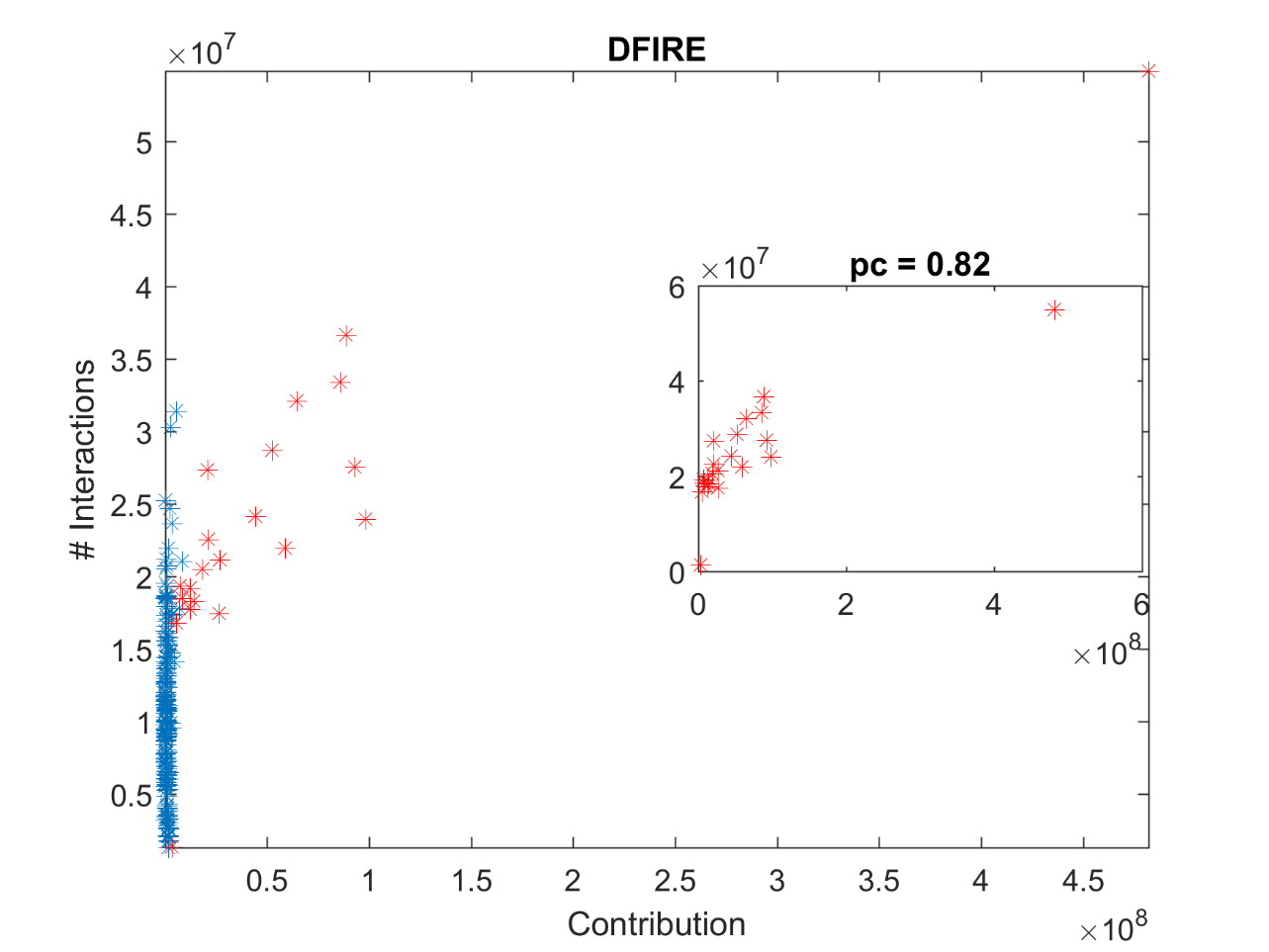


**Figure 6.** The number of pairwise interactions versus their contribution values for DFIRE. The right-hand side inset plot presents the relation between the principal interactions and their associated contribution value.

**
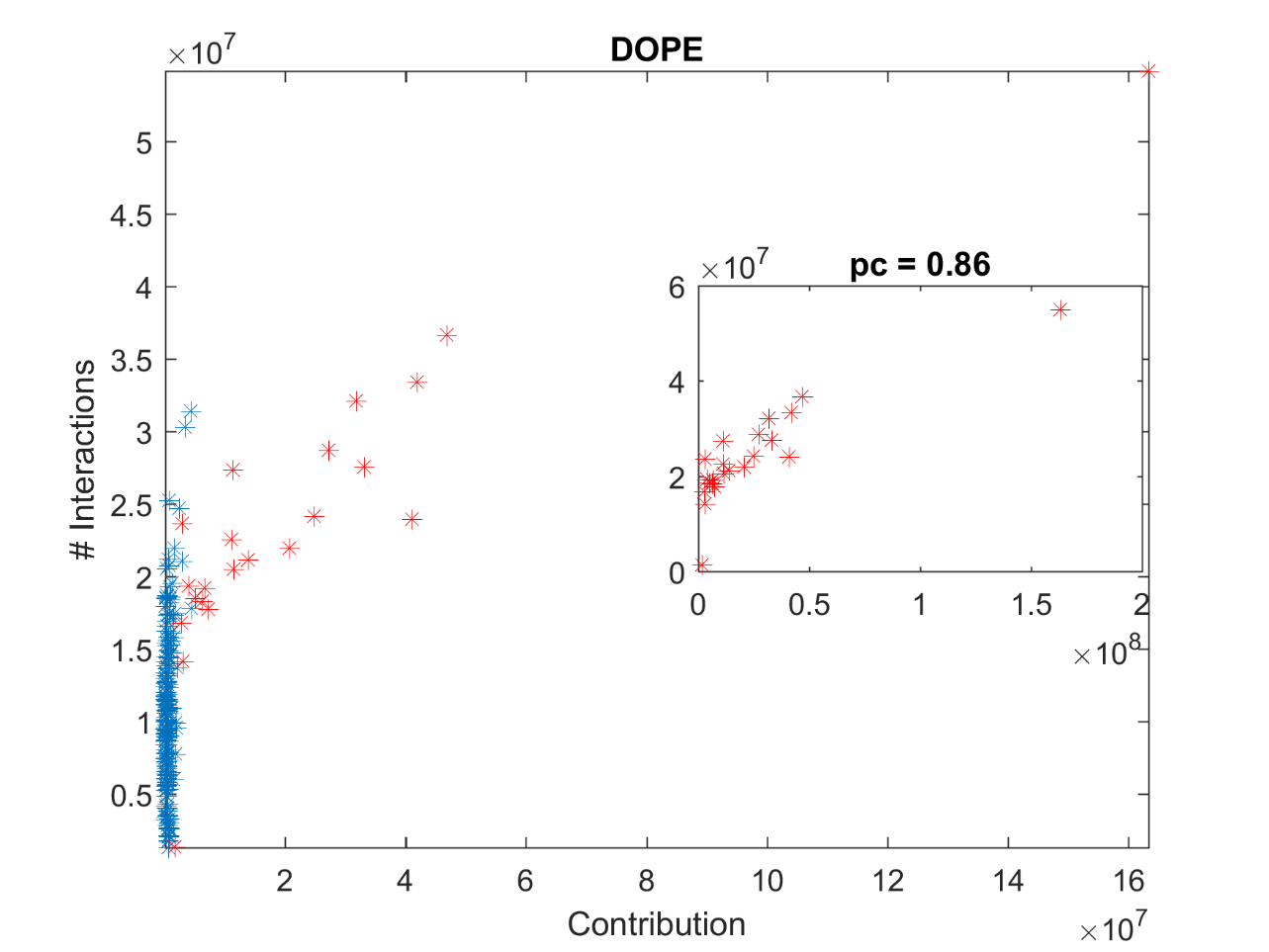
**

**Figure 7.** The number of pairwise interactions versus their contribution values for DOPE. The right-hand side inset plot presents the relation between the principal interactions and their associated contribution value.
